# Combination of gut microbiota and plasma amyloid-β as a potential index for identifying preclinical Alzheimer’s disease: a cross-sectional analysis from the SILCODE study

**DOI:** 10.1186/s13195-022-00977-x

**Published:** 2022-02-14

**Authors:** Can Sheng, Kun Yang, Beiqi He, Wenying Du, Yanning Cai, Ying Han

**Affiliations:** 1 Department of Neurology, Xuanwu Hospital of Capital Medical University, Beijing, 100053 China; 2 Evidence-Based Medicine Center, Xuanwu Hospital of Capital Medical University, Beijing, 100053 China; 3 Department of Epidemiology and Biostatistics, School of Public Health, Capital Medical University, Beijing, 100069 China; 4 Key Laboratory of Biomedical Engineering of Hainan Province, School of Biomedical Engineering, Hainan University, Haikou, 570228 China; 5 Department of Neurobiology, Xuanwu Hospital of Capital Medical University, Beijing, 100053 China; 6 Key Laboratory for Neurodegenerative Diseases of the Ministry of Education, Beijing, 100053 China; 7 Department of Biobank, Xuanwu Hospital of Capital Medical University, Beijing, 100053 China; 8 Center of Alzheimer’s Disease, Beijing Institute for Brain Disorders, Beijing, 100053 China; 9 National Clinical Research Center for Geriatric Diseases, Beijing, 100053 China

**Keywords:** Alzheimer’s disease, Preclinical, Gut microbiota, Amyloid-β, Plasma

## Abstract

**Background:**

Plasma amyloid-β (Aβ) may facilitate identification of individuals with brain amyloidosis. Gut microbial dysbiosis in Alzheimer’s disease (AD) is increasingly being recognized. However, knowledge about alterations of gut microbiota in preclinical AD, as well as whether the combination of plasma Aβ and gut microbiota could identify preclinical AD, remains largely unknown.

**Methods:**

This study recruited 34 Aβ-negative cognitively normal (CN−) participants, 32 Aβ-positive cognitively normal (CN+) participants, and 22 patients with cognitive impairment (CI), including 11 patients with mild cognitive impairment (MCI) and 11 AD dementia patients. All participants underwent neuropsychological assessments and fecal microbiota analysis through 16S ribosomal RNA (rRNA) Illumina Miseq sequencing technique. Meso Scale Discovery (MSD) kits were used to quantify the plasma Aβ_40_, Aβ_42_, and Aβ_42_/Aβ_40_ in CN− and CN+ participants. Using Spearman’s correlation analysis, the associations of global standard uptake value rate (SUVR) with altered gut microbiota and plasma Aβ markers were separately evaluated. Furthermore, the discriminative power of the combination of gut microbiota and plasma Aβ markers for identifying CN+ individuals was investigated.

**Results:**

Compared with the CN− group, the CN+ group showed significantly reduced plasma Aβ_42_ (*p* = 0.011) and Aβ_42_/Aβ_40_ (*p* = 0.003). The relative abundance of phylum *Bacteroidetes* was significantly enriched, whereas phylum *Firmicutes* and class *Deltaproteobacteria* were significantly decreased in CN+ individuals in comparison with that in CN− individuals. Particularly, the relative abundance of phylum *Firmicutes* and its corresponding SCFA-producing bacteria exhibited a progressive decline tendency from CN− to CN+ and CI. Besides, the global brain Aβ burden was negatively associated with the plasma Aβ_42_/Aβ_40_ (*r* = −0.298, *p* = 0.015), family *Desulfovibrionaceae* (*r* = −0.331, *p* = 0.007), genus *Bilophila* (*r* = −0.247, *p* = 0.046), and genus *Faecalibacterium* (*r* = −0.291, *p* = 0.018) for all CN participants. Finally, the combination of plasma Aβ markers, altered gut microbiota, and cognitive performance reached a relatively good discriminative power in identifying individuals with CN+ from CN− (AUC = 0.869, 95% CI 0.782 ~ 0.955).

**Conclusions:**

This study provided the evidence that the gut microbial composition was altered in preclinical AD. The combination of plasma Aβ and gut microbiota may serve as a non-invasive, cost-effective diagnostic tool for early AD screening. Targeting the gut microbiota may be a novel therapeutic strategy for AD.

**Trial registration:**

This study has been registered in ClinicalTrials.gov (NCT03370744, https://www.clinicaltrials.gov) in November 15, 2017.

**Supplementary Information:**

The online version contains supplementary material available at 10.1186/s13195-022-00977-x.

## Introduction

Alzheimer’s disease (AD) is a progressive and irreversible neurodegenerative disorder, causing globally heavy healthcare burden [1]. Increasing evidence implicates that the pathophysiological process of AD begins 15–20 years before the emergence of clinical symptoms [2, 3]. Given the lack of effective strategies available for delaying or preventing the progression of AD, interventions targeting the preclinical stage of AD may offer the best chance for therapeutic success [2, 4]. Currently, preclinical AD is defined by biomarker evidence of AD-related pathological changes in cognitively healthy individuals. Abnormal amyloid positron emission tomography (PET) scan or low cerebrospinal fluid (CSF) amyloid-β (Aβ)_42_ or Aβ_42_/Aβ_40_ ratio are considered as the evidence of Aβ deposition. It is noteworthy that although amyloid PET is gaining attraction in clinical practice, expensive costs and radioactivity limit its wide application. In addition, lumbar puncture is an invasive procedure, and repeated CSF collection is also challenging. Therefore, exploring novel and potentially alternative hallmarks for identifying preclinical AD are needed.

Gut microbiota is considered as a possible susceptibility factor for AD [5]. Accumulating findings support that gut microbiota has the potential to modulate brain function, such as memory and learning [6, 7]. Cross-sectional preclinical and clinical studies provide the insight that altered gut microbial compositions may contribute to the AD pathology [5, 8], and manipulating gut microbiota can attenuate brain Aβ deposition [9, 10]. Previous studies have demonstrated significant alterations of gut microbiota in patients with AD and MCI compared with healthy controls, such as decreased phylum *Firmicutes*, increased family *Enterobacteriaceae* [11–13]. One recent study also reported the decreased anti-inflammatory genus *Faecalibacterium* in individuals with subjective cognitive decline (SCD), providing the preliminary evidence of altered gut microbiota in elderly adults at risk of AD [14]. However, there still exist some issues regarding current AD microbiome studies. Firstly, it is still unclear whether similar alterations of gut microbial compositions occur in the stage of preclinical AD in vivo. Secondly, in previous researches, the diagnosis of AD and MCI patients was based on clinical symptoms and lack of pathophysiological biomarkers. Therefore, in this study, we aimed to investigate the characteristics of gut microbiota in asymptomatic preclinical individuals with biomarker evidence of Aβ deposition.

Moreover, the peripheral blood may be another promising source for screening AD biomarkers. Previous studies have reported the association of changed plasma Aβ with AD [15–18]. Plasma Aβ seems to be a potential hallmark detecting brain Aβ pathological changes [16, 19, 20]. A recent study showed that plasma Aβ_42/40_ ratio had the potential to identify brain Aβ positivity in preclinical AD individuals [20], suggesting that plasma Aβ may be used as a diagnostic tool in routine clinical work. However, previous published studies regarding the correlation between plasma Aβ and AD pathology are contradictory, especially for plasma Aβ_40_ and Aβ_42_.

The main purposes of this study were (1) to characterize the gut microbiota in the preclinical stage of AD, (2) to assess whether plasma Aβ indexes (Aβ_40_, Aβ_42_, and the ratio of Aβ_40_ and Aβ_42_) were changed in preclinical AD, and (3) to investigate the discriminative power of the combined gut microbiota and plasma Aβ indexes in identifying individuals with preclinical AD.

## Materials and methods

### Participants

In the present study, we recruited a total of 66 right-handed Chinese participants, including 34 Aβ-negative cognitively normal (CN−) participants and 32 Aβ-positive cognitively normal (CN+) participants from the Sino Longitudinal Study on Cognitive Decline (SILCODE) [21]. Each participant underwent routine clinical evaluation, standardized neuropsychological assessments, blood sample tests, fecal sample amplicon sequencing, and Aβ-PET scans. To eliminate the potential influence of different lifestyles (e.g., diet, exercise), ethnicities, and regions on gut microbial compositions, all participants recruited in our study were community-dwelling Han nationality older adults who resided in Beijing for a long time. In addition, each participant finished a semi-structured interview to evaluate their lifestyles in detail (Supplementary Table S1). Participants were diagnosed as CN− according to the following criteria: (1) normal performance on a battery of neuropsychological tests, (2) with negative Aβ deposition in amyloid PET, and (3) failure to meet the criteria for MCI and dementia. Participants were defined as CN+ (preclinical AD) when they matched the criteria: (1) normal performance on standardized neuropsychological tests, (2) aggregated Aβ evidence derived from amyloid PET, and (3) failure to meet the criteria for MCI and dementia. The software G*Power 3.1 was used to estimate the sample size in our study (Supplementary Methods).

We also collected the clinical and fecal data of 11 MCI and 11 AD patients from the SILCODE. In our study, patients with MCI and AD were defined as individuals with cognitive impairment (CI). The definition of MCI was in accordance with the criteria proposed by Jak and Bondi in 2014 [22], which met any one of the following three conditions and failed to meet the criteria for dementia: (1) having impaired scores (defined as >1 SD below the age/education-corrected normative means) on both measures in at least one cognitive domain (memory, language, or speed/executive function); (2) having impaired scores in each of the three cognitive domains (memory, language, or speed/executive function); and (3) the Functional Activities Questionnaire (FAQ) ≥ 9. Patients with AD dementia were diagnosed according to the Diagnostic and Statistical Manual of Mental Disorders (fifth edition), and the guidelines for dementia due to AD proposed by the National Institute on Aging and Alzheimer’s Association (NIA-AA) workgroups [23]. The diagnosis of CI patients was mainly based on clinical symptoms, and their pathophysiological features were not confirmed by amyloid PET or CSF markers in this study.

The exclusion criteria included (1) a history of stroke; (2) major depression, with Hamilton Depression Rating Scale (HAMD) score > 24 points; (3) other central nervous system diseases that may cause cognitive impairment, such as Parkinson's disease, tumors, encephalitis and epilepsy; (4) traumatic brain injury; (5) systemic diseases, such as thyroid dysfunction, syphilis and HIV; (6) psychosis or congenital mental developmental delay; (7) a history of using antibiotics, probiotics, prebiotics, or synbiotics within 3 months before fecal sample collection; (8) the use of corticosteroid, immune stimulating medications, and immunosuppressive agents; (9) major gastrointestinal tract surgery in past 5 years; and (10) severe gastrointestinal diseases, such as irritable bowel syndrome, inflammatory bowel disease, severe gastritis, other dysfunction in digestion and absorption, which has been reported to influence gut microbiota.

This study was registered on ClinicalTrials.gov (Identifier: NCT03370744), and research activities were approved by the Medical Research Ethics Committee and Institutional Review Board of Xuanwu Hospital in the Capital Medical University (ID: [2017]046). Each participant needed to provide a written informed consent before participating in study procedures.

### Clinical data collection and neuropsychological assessments

Clinical data, including age, sex, years of education, body mass index (BMI), apolipoprotein E (APOE) genotype, and medical history of hypertension and diabetes, were collected. All participants carried on a battery of standardized neuropsychological tests as follows: (1) memory domain: the Auditory Verbal Learning Test-HuaShan version [AVLT-H] [24], including AVLT-long delayed recall and AVLT-recognition; (2) executive domain: the Shape Trails Test Part A (STT-A) and the Shape Trails Test Part B (STT-B) [25]; (3) language domain: the Animal Fluency Test (AFT) [26] and the 30-item Boston Naming Test (BNT) [27]; (4) global cognitive function: the Montreal Cognitive Assessment-Basic Version (MoCA-B) [28]; (5) daily functional activities: the FAQ; and (6) mood status: the Hamilton Depression Rating Scale (HAMD) and the Hamilton Anxiety Rating Scale (HAMA).

### Fecal sample collection and DNA extraction

Participants were asked to collect a fresh fecal sample in the morning using certain fecal collection containers (SARSTEDT, Germany). All the samples were transferred to the laboratory and stored at −80°C prior to processing. The DNA in each fecal sample was extracted using a QIAamp DNA Stool Mini Kit (Qiagen, Hilden, Germany). The procedures of DNA extraction were conducted under a Class II biologic safety cabinet. Then, the Thermo NanoDrop 2000 spectrophotometer (Thermo Scientific, MA, USA) was used to quantify the concentration of genomic DNA. The DNA integrity and fragment sizes were assessed using 1% agarose gel electrophoresis (AGE). After that, DNA was re-stored at −80°C prior to subsequent analysis.

### 16S rRNA gene amplicon sequencing

The amplicon sequencing procedures were performed in an Illumina Miseq PE250 platform [29]. The V3-V4 region of the bacterial 16S ribosomal RNA (rRNA) gene was selected for the amplification. There were two universal primers linking with indices and sequencing adaptors. The forward primer (5′-3′) was CCTACGGGRSGCAGCAG (341F), and the reverse primer (5′-3′) was GGACTACVVGGGTATCTAATC (806R). Using a KAPA HiFi Hotstart ReadyMix polymerase chain reaction (PCR) kit, the genomic DNA was utilized as a template for PCR amplification. The PCR products were examined using 2% AGE, and gel extraction was conducted by AxyPrep DNA Gel Extraction Kit (Axygen Biosciences, Union City, CA, USA). Subsequently, the concentration of DNA was quantified by the Thermo NanoDrop 2000 spectrophotometer (Thermo Scientific, MA, USA), and the quantity of DNA was assessed using 2% AGE. Finally, sequencing libraries were quantified using Qubit and then pooled to obtain a sufficient concentration.

### Sequence analysis

The sequence analysis procedures were conducted according to our previous study [14]. Paired-end reads were concatenated into longer tags based on the 3′ overlapping regions by VSEARCH (https://github.com/torognes/vsearch). VSEARCH is an open source multithreaded 64-bit tool for processing and preparing amplicon analysis [30]. Then, the primers of merged reads were cut and quality filter was conducted to keep reads error rates less than 1%. After the dereplication, denoised sequences called “zero-radius operational taxonomic units” (ZOTUs) were generated using USEARCH 10 (http://www.drive5.com/usearch/) [31]. Denoising was done by the unoise3 command (http://www.drive5.com/usearch/manual/unoise_algo.html), which was used to identify all correct biological sequences in the reads. Taxonomy was assigned using the Ribosomal Database Project (RDP) as the reference database. After generating the amplicon sequence variants (ASV) table, all samples were normalized to the same number of reads.

Additionally, alpha diversity and beta diversity indexes were calculated based on normalized ASV counts using the online analysis (MicrobiomeAnalyst, https://www.microbiomeanalyst.ca/MicrobiomeAnalyst/home.xhtml). Alpha diversity means the diversity in a single ecosystem or sample. The main metrics of alpha diversity included Chao1, ACE, Shannon, and Simpson in our study. The Chao1 and ACE metrics were used to evaluate the number of ZOTU, which mainly reflected the community richness in a sample. The Channon and Simpson metrics focusing on assessing the community diversity of a sample. Beta diversity were employed to exhibit the different gut microbial communities between different groups. Core microbiota based on the ASV level was also identified by Core microbiome analysis (sample prevalence = 20%, relative abundance = 0.01%) in MicrobiomeAnalyst.

### Plasma Aβ tests

Blood samples (2-ml venous blood) were collected between 7: 00 and 8: 00 in the morning after an overnight fast using EDTA tubes. After repeated centrifugation for 15 min at 4°C (speed: 2500 g/min), supernatants were collected as the plasma. All plasma samples were stored at −80°C and thawed immediately on ice before assaying. In our study, the concentration of plasma Aβ_40_ and Aβ_42_ was quantified using Meso Scale Discovery (MSD) method. V-PLEX Aβ Peptide Panel 1 (4GB): K15199e kits (MSD, Rockville, Maryland, USA) were used. All samples were measured in duplicate using the same aliquot following the manufacturer’s instructions. The inter- and intra-plate coefficient of variation for plasma Aβ_40_ and Aβ_42_ was within 5%. Plasma Aβ_40_, Aβ_42_, and their ratio (Aβ_42_/Aβ_40_) indexes were used for the subsequent analysis.

### Neuroimaging data acquisition

In SILCODE, [^18^F] florbetapir (AV-45) PET and MRI scans were performed on an integrated simultaneous 3.0 T TOF PET/MR scanner (SIGNA PET/MR, GE Healthcare, Milwaukee, Wisconsin, USA) at Xuanwu Hospital of Capital Medical University, Beijing. After an intravenous injection of 7–10 mCi [^18^F] florbetapir radiotracer, participants had a rest for approximately 40 min. Then, a 20-min static PET scan was acquired. The PET data were obtained using a time-of-flight ordered subset expectation maximization (TOF-OSEM) algorithm with the following parameters: 8 iterations, 32 subsets matrix = 192 × 192, field of view (FOV) = 350×350mm^2^, and half-width height = 3. The parameters for T1-weighted 3D brain structural images were as follows: SPGR sequence, FOV = 256 × 256 mm^2^, matrix = 256×256, slice thickness = 1 mm, gap = 0, slice number = 192, repetition time (TR) = 6.9 ms, echo time (TE) = 2.98 ms, inversion time (TI) = 450 ms, flip angle = 12°, and voxel size = 1×1×1 mm^3^.

### Imaging preprocessing and analysis

The [^18^F] florbetapir PET images were preprocessed using the Statistical Parametric Mapping (SPM12) toolbox (http://www.fil.ion.ucl.ac.uk/spm/software/spm12/). PET images were registered to the corresponding T1 images, which were then segmented into GM, white matter, and CSF tissue probability maps. Furthermore, the registered PET images were nonlinearly registered into the Montreal Neurological Institute (MNI) stereotactic template and resampled into 3 × 3 × 3 mm^3^ voxels. Finally, normalized PET images were smoothed by a Gaussian isotropic kernel with an 8 mm full-width at half maximum (FWHM) to improve the signal-to-noise ratio. Global standard uptake value rate (SUVR) of PET scan was calculated as an average of SUVs in the whole brain with the cerebellum as the reference region [32]. Positive Aβ burden was defined when the SUVR was above or equal to 1.18 based on the previous studies [32, 33].

### Statistical analysis

The IBM SPSS Statistics 26.0 and R-3.6.3 were used for the statistical analysis. A Shapiro-Wilk test was used to confirm data normality. Demographic information, neuropsychological assessments, and plasma Aβ indexes were compared using the two-sample *t* test, Mann-Whitney *U* test or Pearson’s chi-squared test as appropriate. Venn diagram was drawn with the R package “VennDiagram”. Mann-Whitney *U* test was performed to compare alpha diversity indexes between the CN− and CN+ group, while Kruskal-Wallis test was used to compare the alpha diversity among the CN−, CN+, and CI groups. Beta diversity was calculated using the principal coordinate analysis (PCoA) and permutational multivariate analysis of variance (PERMANOVA) based on Bray-Curtis index. We also used non-metric multidimensional scaling (NMDS) and analysis of similarities (ANOSIM) to calculate the statistical significance. Linear discriminant analysis (LDA) effect size (LEfSe) method (http://huttenhower.sph.harvard.edu/lefse/) was used to identify differentially abundant taxa between the CN+ and CN− group, with an alpha cutoff of 0.05 and an effect size cutoff of 2.0. The general linear models (GLMs) were further employed to evaluate the differences of these gut microbiota identified by the LEfSe, with age, sex, BMI, and APOE as possible confounding factors. In addition, for taxa with a prevalence ≥1%, we also evaluated taxonomic differences at the phylum, class, order, family, and genus levels using the Mann-Whitney *U* test, with Bonferroni adjustment. Taxonomic differences among CN−, CN+, and CI was calculated using the Kruskal-Wallis test. The associations of global brain SUVR with altered gut microbiota and plasma Aβ markers were separately evaluated using Spearman’s correlation analysis.

To determine whether the combination of altered gut microbiota and plasma Aβ have the potential to distinguish individuals with CN+ from CN−, receiver operating characteristic (ROC) curve and the area under the ROC curve (AUC) were calculated. Multivariable logistic regression models based on gut microbiota with significant group differences and plasma Aβ indexes were separately built in a stepwise manner. The ROC curves were compared with Delong’s statistic method using MedCalc19.0.4 software [34]. Statistical significance was set as *p* < 0.05.

## Results

### Demographic information, neuropsychological assessments, and plasma Aβ

The detailed demographics of all CN participants in this study can be found in Table 1. No significant differences were found in age, sex, years of education, BMI, APOE ε4 carrier, diabetes, hypertension, and emotional status between the two groups (all *p* > 0.05). The CN+ group showed significantly lower score in the AVLT-long delayed recall (*p* = 0.018) and higher score in the STT-B (*p* = 0.039) than that in the CN− group. However, there was no significant difference between the CN− and CN+ groups in other neuropsychological tests, including MoCA-B, AVLT-recognition, STT-A, AFT, BNT, and FAQ (*p* > 0.05). In addition, the characteristics of CI patients (*n* = 22, MCI = 11, AD = 11) are shown in Supplementary Table S2.

**Table 1:** Demographics and neuropsychological assessments for all CN participants

	CN− (***n*** = 34)	CN+ (***n*** = 32)	***P*** value
**Demographic information**			
Age (years)	66.91 ± 5.28	68.44 ± 5.35	0.507
Sex (M/F)	8/26	10/22	0.482
Education (years)	12.76 ± 2.99	12.69 ± 2.62	0.901
BMI	23.78 ± 2.90	24.42 ± 2.74	0.287
APOE ε4 (%)	11 (32.35%)	12 (37.50%)	0.661
Diabetes (%)	5 (14.71%)	1(3.13%)	0.102
Hypertension (%)	15 (44.12%)	12 (37.50%)	0.585
**Neuropsychological tests**			
HAMD	2.88 ± 3.51	3.81 ± 3.83	0.199
HAMA	3.44 ± 3.26	3.75 ± 2.65	0.407
MoCA-B	25.76 ± 2.73	26.28 ± 2.76	0.385
AVLT-D (long)	8.74 ± 1.66	7.75 ± 1.88	0.018*
AVLT-R	22.94 ± 1.13	22.63 ± 1.62	0.489
STT-A	51.47 ± 15.72	62.53 ± 24.66	0.088
STT-B	121.09 ± 32.60	142.22 ± 47.95	0.039*
AFT	18.74 ± 4.59	19.91 ± 3.95	0.272
BNT	25.65 ± 2.86	26.25 ± 2.66	0.342
FAQ	1.00 ± 1.72	0.97 ± 1.12	0.333

*Abbreviations*: *CN−* amyloid-β-negative cognitively normal participants, *CN+* amyloid-β-positive cognitively normal participants, *M* male, *F* female, *BMI* body mass index, *APOE* apolipoprotein E, *HAMD* Hamilton Depression Rating Scale, *HAMA* Hamilton Anxiety Rating Scale, *MoCA-B* Montreal Cognitive Assessment-Basic version, *AVLT-D (long)* Auditory Verbal Learning Test-long delayed recall, *AVLT-R* Auditory Verbal Learning Test-recognition, *STT-A* Shape Trails Test Part A, *STT-B* Shape Trails Test Part B, *AFT* Animal Fluency Test, *BNT* Boston Naming Test, *FAQ* Functional Activities Questionnaire

^*^P < 0.05, comparison between CN− and CN+

**Fig. 1:**
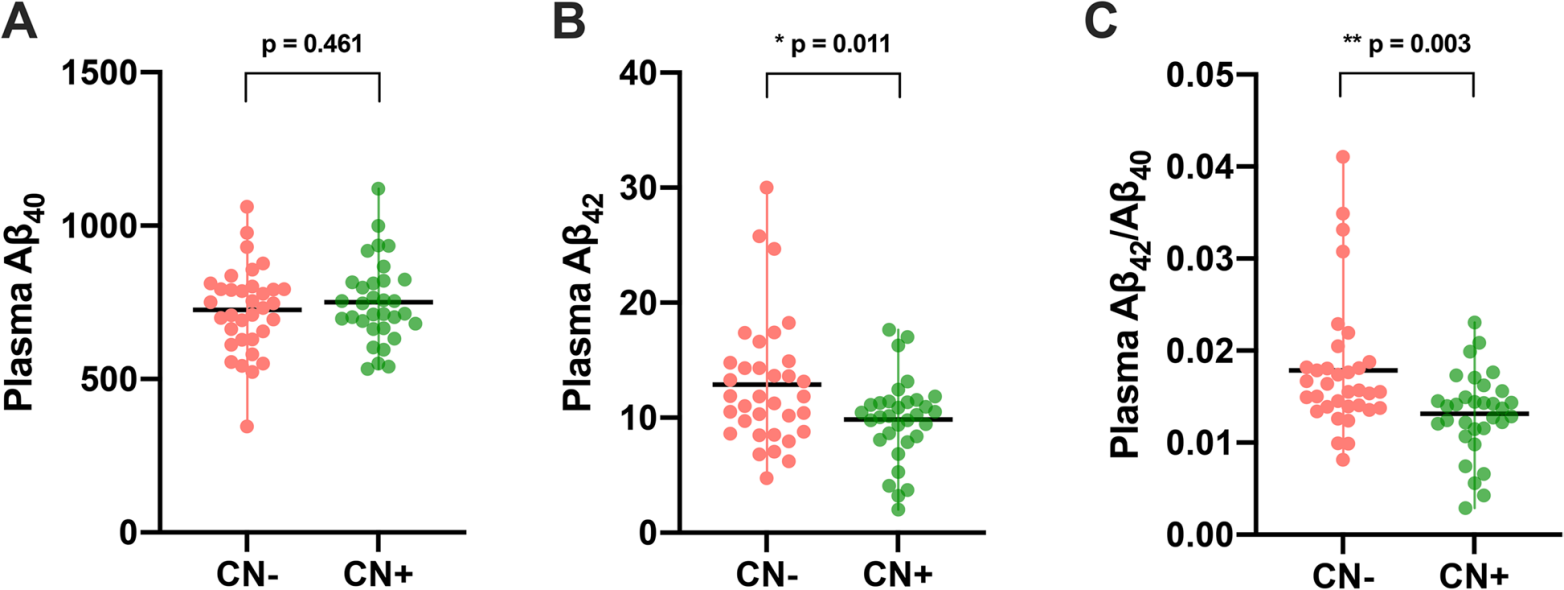
Plasma Aβ levels for CN− and CN+ participants. Scatter plot presented mean with range. No significant difference was observed in plasma Aβ_40_ (**A**), whereas there were significant differences in plasma Aβ_42_ (**B**) and Aβ_42_/Aβ_40_ (**C**) between CN− and CN+. *p < 0.05; **p < 0.01. Aβ amyloid-β; CN−, amyloid-β-negative cognitively normal participants; CN+, amyloid-β-positive cognitively normal participants

Compared with the CN− group, the CN+ group exhibited significantly reduced plasma Aβ_42_ (CN− vs. CN+: 12.97 ± 5.63 vs. 9.84 ± 3.62, *p* = 0.011) and Aβ_42_/Aβ_40_ (CN− vs. CN+: 0.018 ± 0.007 vs. 0.013 ± 0.004, *p* = 0.003), but no significant difference in plasma Aβ_40_ (CN− vs. CN+: 721.98 ± 141.44 vs. 750.48 ± 133.91, *p* = 0.461) (Fig. 1).

### The gut microbiota community profiling

**Fig. 2:**
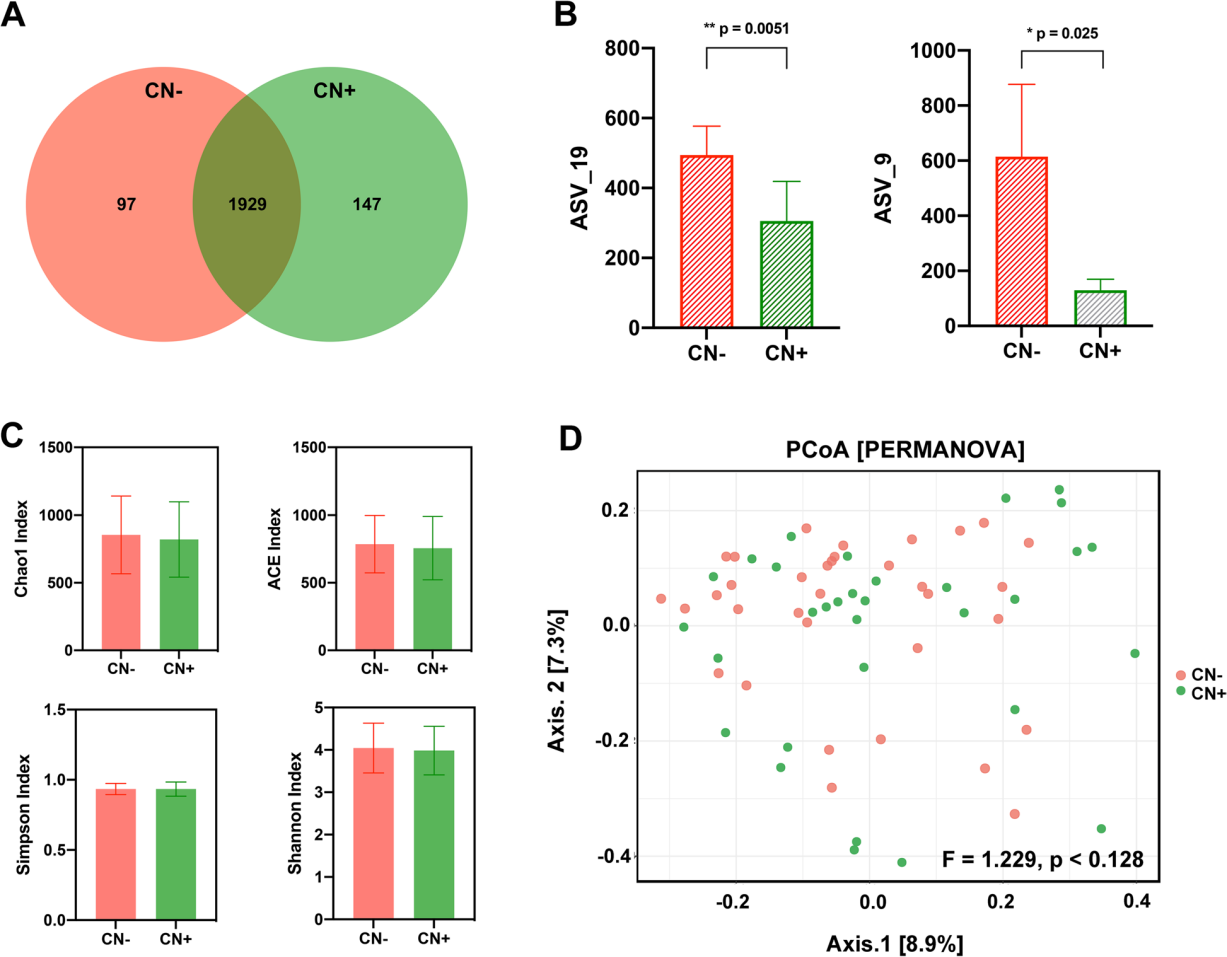
The overall gut microbiota community for CN− and CN+ participants. **A** The Venn diagram illustrated the overlap of ASVs between CN− and CN+, with 1929 ASVs shared in both groups, and 147 ASVs unique for CN+. **B** The key ASVs in both groups. The abundance of ASV_19 and ASV_9 was significantly reduced in CN+ compared with that in CN−. **C** The alpha diversity of gut microbiota between CN− and CN+. Each bar graph represented the mean and standard deviation. There were no significant differences in Chao1, ACE, Simpson, and Shannon indexes between CN− and CN+. **D** The PCoA based on the distribution of ASVs. The structure of gut microbiota in the CN+ group was not significantly different from that in the CN− group. *p< 0.05; **p < 0.01. CN− amyloid-β-negative cognitively normal participants, CN+ amyloid-β-positive cognitively normal participants, ASV amplicon sequence variants, PCoA principal coordinates analysis, PERMANOVA permutational multivariate analysis of variance

As shown in a Venn diagram, the total abundance of ASVs was 2173, and 1929 ASVs were shared in both groups (Fig. 2A). Noteworthy, 147 ASVs were unique to the CN+ group. There were 16 key ASVs between the two groups, and the abundance of two ASVs were significantly lower in the CN+ group than in the CN− group (ASV_19: *p* = 0.0051; ASV_9: *p* = 0.025) (Fig. 2B). Although the four alpha diversity indexes of the CN+ group exhibited a decreased tendency relative to the CN− group, no statistically significant differences were found between the two groups (all *p* > 0.05) (Fig. 2C). The PCoA based on the distribution of ASVs were conducted to illustrate the microbiome space of different samples. However, we found no significant differences in the composition of gut microbiota between the CN− and CN+ groups (PERMANOVA, Bray-Curtis: *F* = 1.229, *p* < 0.128) (Fig. 2D).

We further investigated the alpha diversity and beta diversity among the CN−, CN+, and CI groups. As shown in Figure S1, there was a trend towards increasingly decrease in the Chao1, ACE, and Shannon indexes from CN− to CN+ and CI. Compared with the CN− group, the CI group showed significant decline in the Chao1 (*p* = 0.023) and ACE indexes (*p* = 0.021) (Figure S1A, S1B). Moreover, the NMDS based on ASV distribution showed that the gut taxonomic composition was significantly different between CN− and CI (*R* = 0.097, *p* < 0.009), and the PCoA showed that the difference between the two groups was marginal in statistical significance (*F* = 1.383, *p* < 0.052) (Figure S2A, S2B), suggesting that the fecal microbial structure in CI was significantly different from that of CN−. However, for the beta diversity between CN+ and CI, the PCoA and the NMDS showed no gut taxonomic differences (Figure S2C, S2D).

### The alteration of gut microbial compositions in CN+ participants

**Fig. 3:**
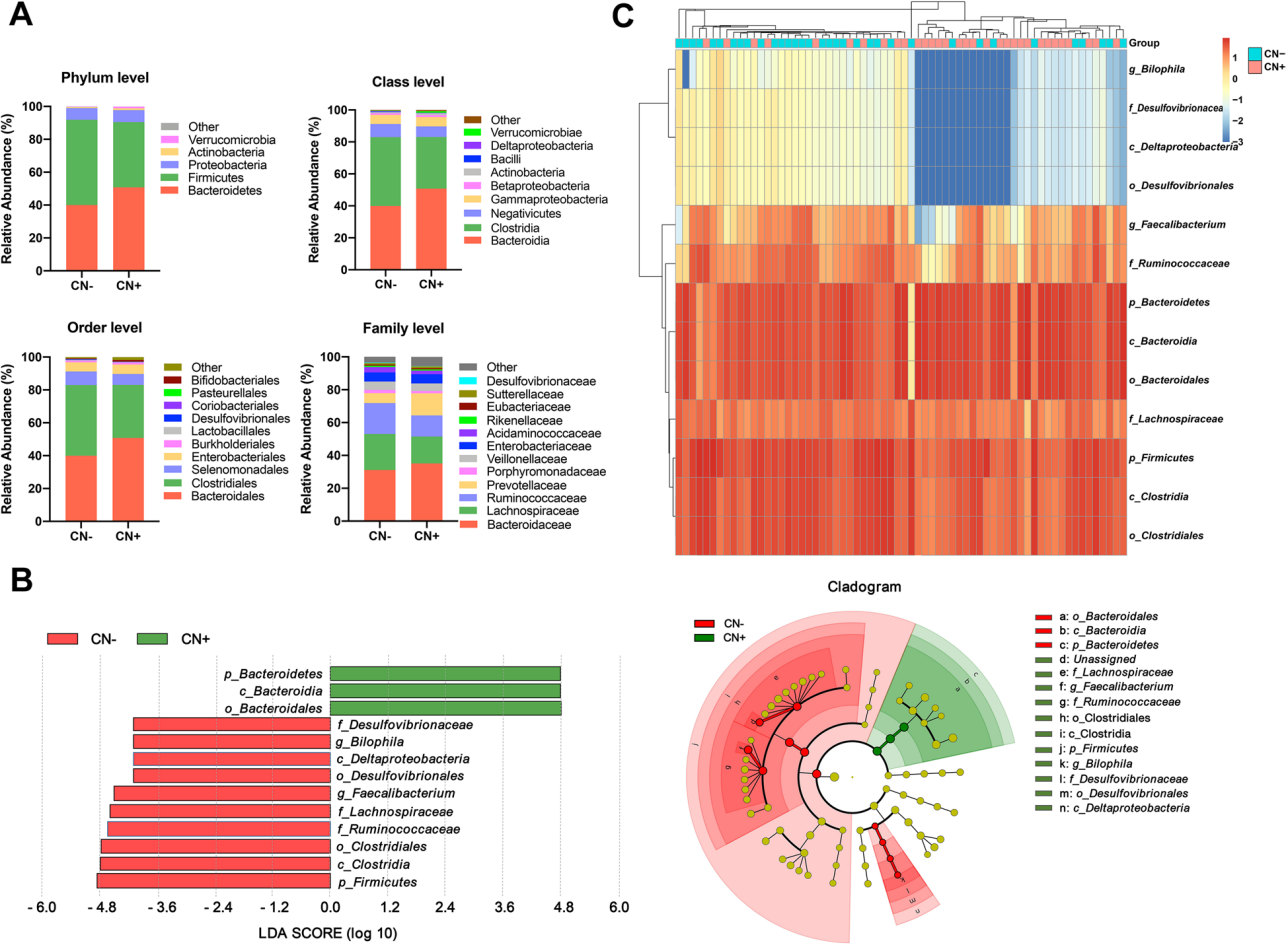
The gut microbial compositions for CN− and CN+ participants. **A** The bacterial community in both groups at different taxonomic levels. Bar graphs indicated the relative abundance of phylum-level, class-level, order-level, and family-level taxa. **B** LEfSe analysis between CN− and CN+. As shown in the histogram of LDA scores for differentially abundant taxa, positive LDA scores indicated the enrichment of taxa in the CN+ group (green), and negative LDA scores indicated the enriched taxa in the CN− group (red). The LDA scores (log10) > 2 and *p* < 0.05 were listed. Cladogram indicated the phylogenetic distribution of gut bacteria. Colors represented different groups (CN−, red; CN+, green). Nodes with different colors represented important taxa in different groups. Yellow nodes suggested no significantly differential taxa between the two groups. **C** The heatmap showing the relative abundance and distribution of differentially abundant taxa identified by the LEfSe method. CN− amyloid-β-negative cognitively normal participants, CN+ amyloid-β-positive cognitively normal participants, LEfSe linear discriminant analysis (LDA) effect size

The overall gut microbial compositions of the CN− and CN+ groups are shown in Fig. 3A at different taxonomic levels. At the phylum level, the predominant bacteria in each group were *Bacteroidetes*, *Firmicutes*, and *Proteobacteria*, followed by *Actinobacteria*. At the class level, *Bacteroidia*, *Clostridia*, *Negativicutes*, *Gammaproteobacteria*, and *Betaproteobacteria* were dominant bacteria. At the order level, there were five dominant bacteria, including *Bacteroidales*, *Clostridiales*, *Selenomonadales*, *Enterobacteriales*, and *Burkholderiales*. At the family level, the dominant bacteria included *Bacteroidaceae*, *Lachnospiraceae*, *Ruminococcaceae*, and *Prevotellacea*. Above all, the predominant gut microbial formation in CN+ was almost consistent with that in CN−.

LEfSe analysis was used to identify the distinct microbiota of CN+ participants. The results showed that the relative abundance of phylum *Bacteroidetes*, class *Bacteroidia*, and order *Bacteroidales* were significantly enriched, whereas phylum *Firmicutes*, class *Clostridia*, class *Deltaproteobacteria*, order *Clostridiales*, order *Desulfovibrionales*, family *Lachnospiraceae*, family *Desulfovibrionaceae*, family *Ruminococcaceae*, genus *Bilophila*, and genus *Faecalibacterium* were significantly reduced in the CN+ group (LDA score cutoff > 2.0, Fig. 3B). The relative abundance and distribution of these selected microbiota were presented in a heatmap (Fig. 3C).

In addition, the GLMs were also used to evaluate the differences of these gut microbiota identified by the LEfSe. We found that no significant differences were found in family *Ruminococcaceae* (*p* = 0.067) and genus *Bilophila* (*p* = 0.177) after controlling for age, sex, BMI, and APOE (Table 2). For taxa with the relative abundance ≥ 1%, the relative abundance of family *Ruminococcaceae* and genus *Faecalibacterium* were not significant after correction for multiple comparisons (*p* > 0.05), whereas abundant differences of order *Bacteroidales* and family *Lachnospiraceae* were marginal in statistical significance (*p* = 0.070 and *p* = 0.066, respectively) (Supplementary Table S3). Meanwhile, no significant interaction effect was found with APOE genotype and the Aβ status in the altered gut microbiota for CN− and CN+ participants (Supplementary Table S4).

**Table 2:** Alterations of the gut microbiota between CN− and CN+ using GLM

	CN− (***n*** = 34)	CN+ (***n*** = 32)	***F***_**(1, 60)**_	***P*** value
p_Bacteroidetes	40.01 ± 16.14	50.76 ± 18.25	7.103	0.010
p_Firmicutes	51.85 ± 16.08	39.71 ± 17.26	7.768	0.007
c_Bacteroidia	40.00 ± 16.15	50.76 ± 18.25	7.109	0.010
c_Clostridia	42.93 ± 15.01	32.26 ± 16.71	5.992	0.017
c_Deltaproteobacteria	0.39 ± 0.51	0.15 ± 0.25	4.472	0.039
o_Bacteroidales	40.00 ± 16.15	50.76 ± 18.25	7.109	0.010
o_Clostridiales	42.93 ± 15.01	32.26 ± 16.71	5.992	0.017
o_Desulfovibrionales	0.39 ± 0.51	0.15 ± 0.25	4.472	0.039
f_Lachnospiraceae	21.92 ± 9.50	16.33 ± 7.19	6.096	0.016
f_Ruminococcaceae	18.86 ± 11.79	12.93 ± 11.82	3.491	0.067
f_Desulfovibrionaceae	0.39 ± 0.51	0.15 ± 0.25	4.472	0.039
g_Bilophila	0.29 ± 0.50	0.13 ± 0.24	1.863	0.177
g_Faecalibacterium	11.41 ± 8.65	7.08 ± 7.99	4.008	0.050

Statistical analysis was conducted using GLM, with age, sex, BMI, and APOE as possible confounding factors

*Abbreviations*: *GLM* general linear model, *CN*− amyloid-β-negative cognitively normal participants, *CN+* amyloid-β-positive cognitively normal participants, *CI* cognitive impairment, *p* phylum, *c* class, *o* order, *f* family, *g* genus

Furthermore, the relative abundance of altered gut microbiota among the CN−, CN+, and CI groups are shown in Figure S3. We found that the phylum *Firmicutes* and its corresponding class *Clostridia*, order *Clostridiales*, family *Lachnospiraceae*, family *Ruminococcaceae*, genus *Lachnospiracea_incertae_sedis*, and genus *Faecalibacterium* taxa showed a progressive decline from CN− to CN+ and CI.

### The association of plasma Aβ markers and gut microbiota with brain Aβ burden

**Fig. 4:**
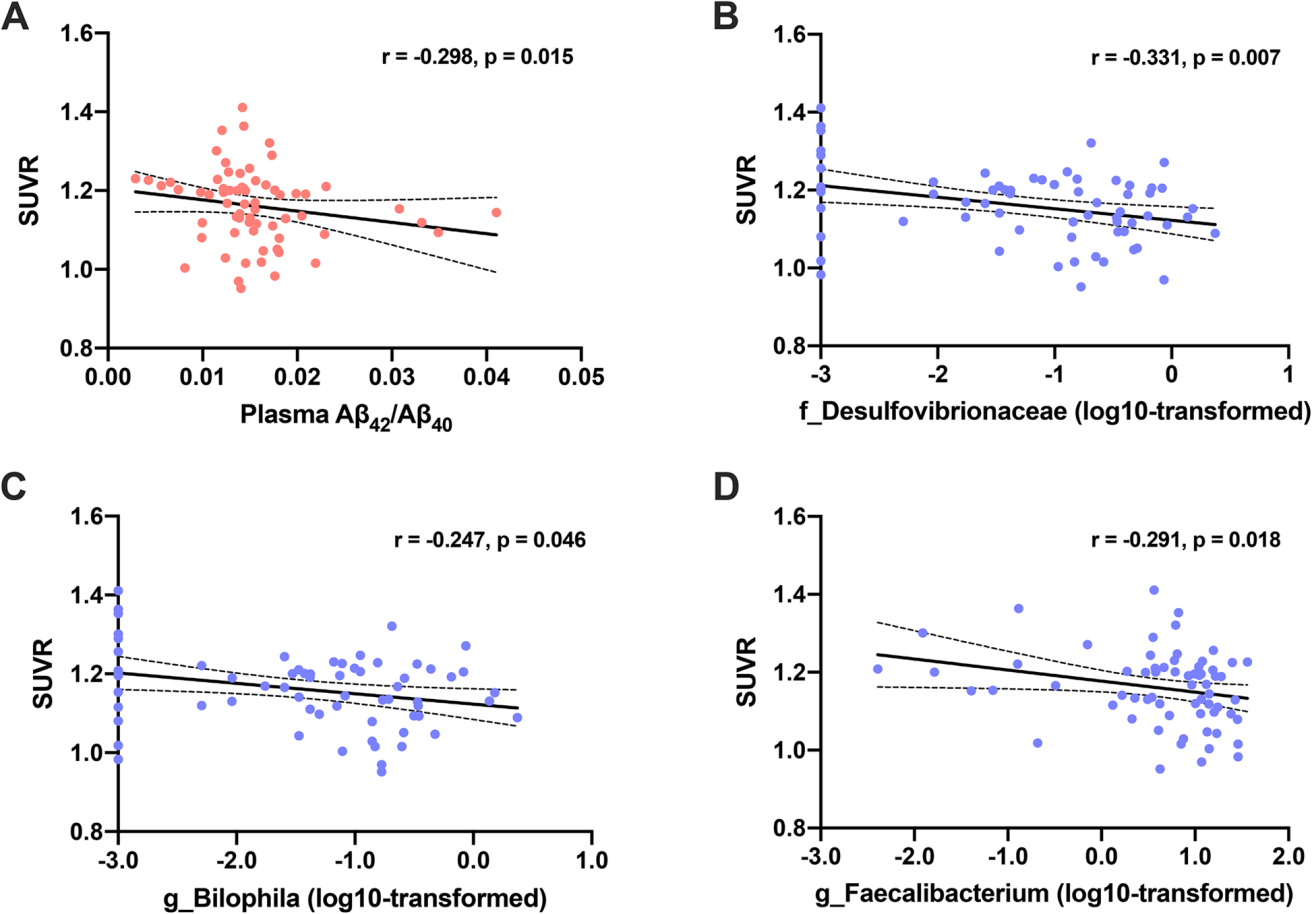
The association of plasma Aβ markers and gut microbiota with global brain Aβ burden. **A** There was negative correlation between global SUVR and plasma Aβ_42_/Aβ_40_. The family *Desulfovibrionaceae* (**B**), genus *Bilophila* (**C**), and genus *Faecalibacterium* (**D**) were negatively correlated with the global SUVR. Aβ amyloid-β, SUVR standard uptake value rate

In our study, we found that the global brain SUVR was negatively associated with plasma Aβ_42_/Aβ_40_ for all CN participants (*r* = −0.298, *p* = 0.015) (Fig. 4A). However, no significant association with plasma Aβ_42_ and Aβ_40_ was observed (*r* = −0.209, *p* = 0.093; *r* = −0.085, *p* = 0.499, respectively). Subsequently, we investigated the association of altered gut microbiota with the brain Aβ burden. As shown in Fig. 4B–D, the log10-transformed family *Desulfovibrionaceae*, genus *Bilophila*, and genus *Faecalibacterium* was negatively correlated with the global brain SUVR (*r* = −0.331, *p* = 0.007; *r* = −0.247, *p* = 0.046; *r* = −0.291, *p* = 0.018, respectively).

### The discriminative power of the combined gut microbiota and plasma Aβ

**Fig. 5:**
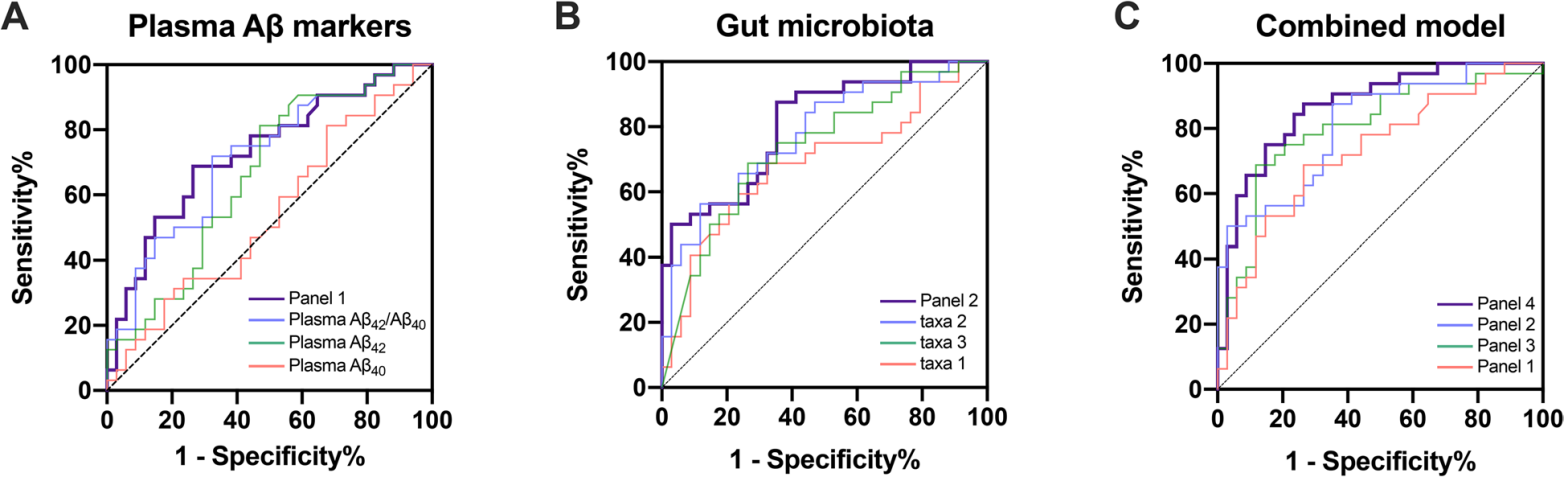
ROCs for CN+ participants. **A** The discriminative power of individual plasma Aβ markers and the combined panel in identifying CN+ from CN−; **B** The discriminative power of each of the gut taxa and the combined panel in identifying CN+ from CN−; **C** The predicted values of the combined panels in identifying CN+ from CN−. Aβ, amyloid-β; CN−, amyloid-β-negative cognitively normal participants; CN+, amyloid-β-positive cognitively normal participants; panel 1, the combined plasma Aβ_40_, Aβ_42_, and Aβ_42_/ Aβ_40_; panel 2, the combined taxa 1, taxa 2, and taxa 3; panel 3, the combined clinical cognitive tests (including MoCA-B, AVLT-long delayed recall, AVLT-R, STT-A, STT-B, AFT, BNT); panel 4, the combined plasma Aβ markers, gut taxa, and cognitive tests

Using the ROC analysis approach, we first estimated the discriminative power of each of the plasma Aβ markers in identifying individuals with CN+ from CN−. The plasma Aβ_42_/Aβ_40_ showed a relatively good discriminative power, followed by the plasma Aβ_42_, with AUCs of 0.715 (95%: 0.591 ~ 0.838) and 0.660 (95%: 0.528 ~ 0.792), respectively. At the cutoff optimized for balanced sensitivity and specificity (Youden’s Index), the sensitivity of all single plasma markers was between 71.88% and 81.25%, whereas the specificity was 52.94% and 67.65% for plasma Aβ_42_ and Aβ_42_/Aβ_40_, respectively, and only 32.25% for plasma Aβ_40_. Then, the combination of the plasma Aβ_40_, Aβ_42_, and Aβ_42_/Aβ_40_ further improved the discriminative power (*panel 1*: AUC = 0.730, 95%: 0.608 ~ 0.852; Table 3; Fig. 5A).

**Table 3:** The AUC and sensitivity and specificity at Youden’s cutoff to identify CN+ participants

	AUC (95% CI)	Youden’s cut point	Sensitivity (%)	Specificity (%)	***P*** value
**Plasma Aβ markers**					
Plasma Aβ_40_	0.539 (0.399 ~ 0.679)	663.7 pg/mL	81.25	32.35	0.586
Plasma Aβ_42_	0.660 (0.528 ~ 0.792)	11.69 pg/mL	81.25	52.94	0.025
Plasma Aβ_42_/Aβ_40_	0.715 (0.591 ~ 0.838)	0.015	71.88	67.65	0.003
***Panel 1***	0.730 (0.608 ~ 0.852)	0.510	68.75	73.53	0.001
**Gut microbiota**					
Taxa1	0.686 (0.555 ~ 0.817)	0.500	68.75	67.65	0.009
Taxa2	0.775 (0.663 ~ 0.887)	0.625	56.25	88.24	< 0.001
Taxa3	0.734 (0.613 ~ 0.855)	0.574	68.75	73.53	0.001
***Panel 2***	0.810 (0.707 ~ 0.912)	0.383	87.50	64.71	< 0.001
**Cognitive scores**					
***Panel 3***	0.802 (0.691 ~ 0.912)	0.573	68.75	88.24	< 0.001
**Combined model**					
***Panel 4***	0.869 (0.782 ~ 0.955)	0.356	87.50	73.53	< 0.001

Taxa 1, combined phylum *Bacteroidetes*, class *Bacteroidia*, and order *Bacteroidales*

Taxa 2, combined phylum *Firmicutes*, class *Clostridia*, order *Clostridiales*, family *Lachnospiraceae*, family *Ruminococcaceae*, and genus *Faecalibacterium*

Taxa 3, combined class *Deltaproteobacteria*, order *Desulfovibrionales*, family *Desulfovibrionaceae*, and genus *Bilophila*

Panel 1, the combined plasma Aβ_40_, Aβ_42_, and Aβ_42_/Aβ_40_

Panel 2, the combined taxa 1, taxa 2, and taxa 3

Panel 3, the combined clinical cognitive tests (MoCA-B, AVLT-long delayed recall, AVLT-R, STT-A, STT-B, AFT, BNT)

Panel 4, the combined plasma Aβ markers, gut taxa, and cognitive tests

*Abbreviations*: *AUC* area under curve, *Aβ* amyloid-β, *CN+* amyloid-β-positive cognitively normal participants

We also calculated the classification power of each of the gut taxa, as well as that of the combined gut taxa for distinguishing CN+ from CN−. We defined the combined phylum *Bacteroidetes*, class *Bacteroidia*, and order *Bacteroidales* as the taxa 1; the combined phylum *Firmicutes*, class *Clostridia*, order *Clostridiales*, family *Lachnospiraceae*, family *Ruminococcaceae*, and genus *Faecalibacterium* as the taxa 2; the combined class *Deltaproteobacteria*, order *Desulfovibrionales*, family *Desulfovibrionaceae*, and genus *Bilophila* as the taxa 3. Compared with the single taxa, the combination of the taxa1, taxa2, and taxa3 (*panel 2*) showed relatively higher classification accuracy, with an AUC of 0.810 (95%: 0.707 ~ 0.912; Table 3; Fig. 5B). In addition, the discriminative power of the combined cognitive tests (including MoCA-B, AVLT-long delayed recall, AVLT-R, STT-A, STT-B, AFT, BNT, *panel 3*) was relatively good, with an AUC of 0.802 (95%: 0.691 ~ 0.912; Table 3). Finally, as is shown in Fig. 5C and Table 3, compared with the *panel 1*, the AUC of the *panel 4* improved to 0.869 (95% CI 0.782 ~ 0.955) after adding the *panel 2* and *panel 3* (*p* = 0.0086, DeLong’ test), suggesting that the combination of plasma markers, gut microbiota, and cognition reached an optimal classification.

## Discussion

In this study, we characterized the gut microbiota in the preclinical stage of AD and further investigated the potential classification efficiency of the combined gut microbiota and plasma Aβ markers for identifying CN individuals with brain amyloidosis. We found that plasma Aβ_42_ and Aβ_42_/Aβ_40_ significantly reduced in the CN+ group relative to the CN− group. The relative abundance of phylum *Bacteroidetes* were enriched, whereas taxa in *Firmicutes* and *Proteobacteria* phyla were reduced in CN+. In addition, the global brain, Aβ burden was negatively associated with the plasma Aβ_42_/Aβ_40_, family *Desulfovibrionaceae*, genus *Bilophila* and genus *Faecalibacterium* for all CN participants. Importantly, the combination of plasma Aβ markers, altered gut microbiota, and clinical cognition showed the potential of distinguishing CN+ from CN−, suggesting that the combined gut microbiota and plasma Aβ markers may serve as a minimally invasive and cost-effective index for screening preclinical AD.

In the present study, we found that the level of plasma Aβ_42_ and Aβ_42_/Aβ_40_ in CN+ was lower than that in CN−. Besides, plasma Aβ_42_/Aβ_40_ was negatively associated with brain Aβ burden, whereas plasma Aβ_42_ and Aβ_40_ showed no correlation with brain amyloidosis. Our results indicated that the ratio of Aβ_42_ and Aβ_40_ appeared to be more predictive of brain Aβ pathological changes than Aβ_42_ and Aβ_40_. Currently, the published results regarding the correlation between plasma Aβ and AD are conflicting. Many studies evaluated plasma Aβ_42_ as a biomarker of AD [15, 16, 35, 36], whereas a systematic review and meta-analysis comprising 231 articles reported that plasma Aβ_42_ and Aβ_40_ were not associated with AD [37]. However, recent reports have demonstrated that plasma Aβ_42_/Aβ_40_ are strongly predictive of brain amyloidosis, even in individuals with CN+ [20, 36], which was consistent with our findings. Schindler also pointed out that plasma Aβ_42_/Aβ_40_ had higher concordance with brain amyloidosis than plasma Aβ_42_ and Aβ_40_ separately. One possible explanation is that Aβ_42_/Aβ_40_ may normalize for preanalytical variability or differences in Aβ levels related to circadian rhythms or other biological variation not related to brain amyloidosis. In the current study, plasma Aβ markers, especially Aβ_42_/Aβ_40_, had the potential to distinguish CN+ individuals from CN− (AUC = 71.5%). This accuracy is highly comparable to what was reported in another study that the diagnostic accuracy of Aβ_42_/Aβ_40_ measurement alone for amyloid PET positivity was reasonably good with an AUC of 73.0% [38]. Since the changed plasma Aβ levels are rather small, we thought that the combination of multiple markers may assist in achieving a better discriminative power between CN+ and CN−. Therefore, a model including Aβ_42_/Aβ_40_, Aβ_42_ and Aβ_40_ improved the diagnostic efficiency, with an AUC of 73.0%.

In our study, individuals with CN+ showed similar gut microbial alterations like AD, suggesting that changes of the gut microbial profiling occurred in the preclinical AD. Previous studies have demonstrated markedly decreased phylum *Firmicutes* in AD patients compared with healthy controls, which was similar to our findings. To the best of our knowledge, phylum *Firmicutes* is responsible for the regulation of inflammatory responses and human metabolic functions, which may in turn affect behavior and cognition [39, 40]. Accumulating evidence has indicated that intestinal dysbiosis has an adverse impact on the human neuroinflammation, which further contributes to the occurrence and progression of AD [41–43]. The depletion of phylum *Firmicutes* may promote the production of pro-inflammatory cytokines and toxic metabolites, and meanwhile, reduce the quantity of beneficial substances such as short-chain fatty acids (SCFAs), leading to the damage of gut epithelial barrier and subsequent blood–brain barrier (BBB) dysfunctions [44]. In addition, the relative abundance of class *Clostridia*, order *Clostridiales*, family *Ruminococcaceae* and family *Lachnospiraceae*, which is key SCFA-producing bacteria belonging to phylum *Firmicutes*, was also significantly reduced in CN+ participants. Current studies based on animal models have suggested the inner correlation between the decreased gastrointestinal SCFA level and the onset of AD [45]. SCFAs are the gut microbial-derived metabolites, which are mainly from dietary components that are incompletely hydrolyzed due to a lack of appropriate enzymes [6, 46]. SCFAs may be strongly involved in glucose regulation in humans and have beneficial effects on energy homeostasis and metabolism [47, 48]. Researchers have proposed that SCFAs play a critical role in the maintenance of homeostasis within the central nervous system [49] and ameliorating the BBB permeability [50]. The family *Ruminococcaceae* and family *Lachnospiraceae* are also related to insulin resistance, which is regarded as a high-risk factor for developing AD [51]. In this study, the results also revealed that genus *Faecalibacterium* was negatively correlated with brain Aβ deposition. To our knowledge, the genus *Faecalibacterium* and its metabolites butyrate have anti-inflammatory effects. The reduced genus *Faecalibacterium* may lead to the decreased anti-inflammatory role, which further results in AD pathology. It is noteworthy that there was a progressively declined trend in phylum *Firmicutes*, as well as its corresponding class, order, family, and genus taxa from CN− to CN+ and CI, indicating again that the gut microbial alterations in preclinical AD might be at an intermediate stage in the AD continuum.

We also found the significantly enriched phylum *Bacteroidetes* and its relatives in CN+ participants, but no progressively increased *Bacteroidetes* in CI patients when compared to CN− individuals. The previously published literatures regarding the association of *Bacteroidetes* with AD are contradictory. For instance, Nicholas M. Vogt et al. reported significantly increased bacterial abundance in *Bacteroidetes* [12], whereas Zhuang and his colleagues found a mild decrease in the abundance of *Bacteroidetes* among AD patients [52]. Interestingly, one study characterizing the gut microbiota in the prodromal stage of AD found that *Bacteroidetes* was significantly enriched in amnestic MCI patients and unexpectedly decreased in AD patients to the normal level [11]. Likewise, in our study, individuals in the preclinical stage of AD showed the highest abundance in phylum *Bacteroidetes* among the CN−, CN+, and CI groups. The phylum *Bacteroidetes* encompasses a diverse group of gram-negative commensal bacteria in the gut [53], whose major outer membrane component is lipopolysaccharide (LPS). LPS plays a crucial role in triggering systemic inflammation and the release of pro-inflammatory cytokines, which can further lead to brain amyloid deposition [54, 55]. Thus, the enriched phylum *Bacteroidetes and* its relatives in CN+ may result in increased translocation of LPS from the gut to systemic circulation, which in turn may exacerbate AD pathology through inflammation or other mechanisms [12].

Moreover, class *Deltaproteobacteria*, as well as its corresponding order, family, and genus taxa were significantly decreased in CN+ individuals. Class *Deltaproteobacteria* is the fourth described class of the phylum *Proteobacteria*, including a series of sulfate-reducing bacteria [53, 56]. The metabolic end-product of these bacteria is the hydrogen sulfide, the overproduction of which in the gastrointestinal tract has been linked to ulcerative colitis and colon cancer [57]. Additionally, in our study, we also found negative associations of class *Deltaproteobacteria*, order *Desulfovibrionales*, family *Desulfovibrionaceae*, genus *Bilophila* with brain Aβ burden, possibly providing a clue that *Deltaproteobacteria* may contribute to AD pathology. However, the correlation between *Deltaproteobacteria* and AD is largely unclear. A prior study reported the enriched proteobacteria in AD and MCI patients compared with healthy controls, which focused on class *Gammaproteobacteria* and family *Enterobacteriaceae* [11]. The discrepancy of different studies may be attributed to differences in sample size, population, RNA sequencing method, and comorbidity condition.

In this study, we also assessed the discriminative power of these altered gut microbiota. The taxa 2 showed a relatively good discriminative power, followed by the taxa 3 and taxa 1, with AUCs of 0.775, 0.734, and 0.686, respectively. Furthermore, the combination of three taxa showed a relatively higher classification accuracy, with an AUC of 0.810. When we underwent the simultaneous evaluation of plasma Aβ markers, gut microbiota, and clinical cognitive scores, the discriminative power reached a larger diagnostic accuracy for CN+ individuals (AUC = 0.869), suggesting that combination of plasma Aβ and gut microbiota provides a potentially valuable tool for the identification of amyloid PET status.

Our study showed a very slight non-significant trend of decreased alpha diversity in CN+ compared with that in CN−. After adding the CI group, the alpha diversity expressed as Chao1 and ACE was lower in CI than in CN−, which was in agreement with the previous studies [12]. Nevertheless, in one study by Liu et al., although the whole alpha diversity also decreased in AD, indexes with statistical significance mainly in Shannon and Simpson [11]. Moreover, the Chao1, ACE, and Shannon indexes of CN+ individuals were at an intermediate stage between CN− and CI in our study. Notably, the pathophysiological mechanism of altered alpha diversity still needs further investigation. For the beta diversity, the gut taxonomic composition of CI was significantly different from that of CN− using the NMDS, while there were no gut taxonomic differences between CN− and CN+, and between CN+ and CI. Our findings were consistent with the previous reports that the fecal microbial structure in AD was significantly different from that of controls [11, 12, 52].

### Limitations

Here, there are also some limitations which warrant attention. Firstly, this is a preliminary, single-center study and the sample size is relatively small. In future studies, a larger sample size from multiple centers is essential to provide more evidences. Secondly, although the participants in our study have been matched in the demography, nationality, place of residence and lifestyles, the effect of other potential factors (e.g., medications, external stressors, immune function) is still difficult to control. Cryan et al. consider that most of the current gut-related studies are underpowered, with participant-selection bias, inconsistent sample size, different sequencing protocols, bioinformatics pipelines, and statistical methods [5]. Thus, to objectively mirror the intrinsic relation of the microbiota-gut-brain axis, more standardized and well-designed studies are needed in the future. Thirdly, in our study, not all the CI patients had amyloid-PET data, and the diagnosis of MCI and AD was mainly based on the clinical practice [58, 59]. Therefore, in the future, CI patients with evidence of brain amyloidosis are necessary to be recruited to provide more accurate evidence of the gut microbiota in the spectrum of AD. Finally, 16S rDNA amplicon sequencing analysis can only reach genus-level resolution, and it is more sensitive to the specific primers and number of PCR cycles chosen. Metagenomic sequencing analysis, characterized by extending taxonomic resolution to the species- or strain-level and simultaneously providing potential functional information, will provide more microbial information [60]. In the future work, the combination of the 16S rDNA amplicon sequencing and metagenomic sequencing techniques can be used.

## Conclusions

In summary, this study characterized the gut microbiota in individuals with preclinical AD and further illuminated the association of gut microbiota and plasma Aβ with brain amyloidosis. Our findings supported that the combination of gut microbiota and plasma Aβ_42_/Aβ_40_ may be used as a screening tool for preclinical AD and targeting gut microbiota may offer novel thoughts towards the therapeutic strategies of AD-related cognitive decline.

## Supplementary Information

**Additional file 1 **: **Supplementary Table S1.** The items of the semi-structured interview for lifestyles. **Supplementary Table S2.** Demographic information and neuropsychological assessments for CI patients. **Supplementary Table S3.** Gut microbial differences between CN- and CN+ with correction for multiple comparisons. **Supplementary Table S4.** The interaction effect between APOE and diagnosis in the altered gut microbiota for CN- and CN+ participants. **Supplementary Figure S1.** The alpha diversity of gut microbiota among the CN-, CN+, and CI groups. Each bar graph represented the mean and standard deviation. The Chao1 and ACE indexes showed significantly decline in CI compared with CN-. There were no significant differences in Simpson and Shannon indexes among the three groups. *, *p* < 0.05. CN-, amyloid-β negative cognitively normal participants; CN+, amyloid-β positive cognitively normal participants; CI, cognitive impairment participants. **Supplementary Figure S2.** The PCoA and NMDS based on the distribution of ASVs. (A) PCoA showed that the difference of gut taxonomic composition between CN- and CI was marginal in statistical significance (F = 1.383, *p* < 0.052); (B) NMDS showed that the gut taxonomic composition was significantly different between CN- and CI (R = 0.097, *p* < 0.009); (C) PCoA showed that the gut taxonomic composition between CN+ and CI was not significantly different (F = 0.850, *p* < 0.712); (D) NMDS showed that the structure of gut microbiota in the CN+ group was not significantly different from that in the CI group (F = -0.020, *p* < 0.698). CN-, amyloid-β negative cognitively normal participants; CN+, amyloid-β positive cognitively normal participants; CI, cognitive impairment participants; ASV, amplicon sequence variants; PCoA, principal coordinates analysis; PERMANOVA, permutational multivariate analysis of variance; NMDS, non-metric multidimensional scaling; ANOSIM, analysis of similarities. **Supplementary Figure S3.** The relative abundance of altered gut microbiota at different taxonomic levels among the CN-, CN+ and CI groups. Bar graphs indicated the relative abundance of phylum-level (A), class-level (B), order-level (C), family-level (D), and genus-level (E) taxa. The phylum *Firmicutes* and its corresponding class *Clostridia*, order *Clostridiales*, family *Desulfovibrionaceae*, family *Ruminococcaceae*, genus *Lachnospiracea_incertae_sedis* and genus *Faecalibacterium* taxa showed a progressive decline from CN- to CN+ and CI. CN-, amyloid-β negative cognitively normal participants; CN+, amyloid-β positive cognitively normal participants; CI, cognitive impairment participants.

## Data Availability

All microbiome sequence data are available upon request from the authors.

## Abbreviations

AD: Alzheimer’s disease
Aβ: Amyloid-β
AFT: Animal Fluency Test
AGE: Agarose gel electrophoresis
APOE: Apolipoprotein E
ASV: Amplicon sequence variants
AVLT-H: Auditory Verbal Learning Test-HuaShan version
BMI: Body mass index
BNT: Boston Naming Test
CN: Cognitively normal
CSF: Cerebrospinal fluid
FAQ: Functional Activities Questionnaire
HAMA: Hamilton Anxiety Rating Scale
HAMD: Hamilton Depression Rating Scale
LEfSe: Linear discriminant analysis LDA effect size
LPS: Lipopolysaccharide
MCI: Mild cognitive impairment
NIA-AA: National Institute on Aging and Alzheimer’s Association
MoCA-B: Montreal Cognitive Assessment-Basic Version
MSD: Meso Scale Discovery
PCoA: Principal coordinate analysis
PCR: Polymerase chain reaction
PERMANOVA: Permutational multivariate analysis of variance
PET: Positron emission tomography
RDP: Ribosomal Database Project
SCD: Subjective cognitive decline
SCFAs: Short-chain fatty acids
SILCODE: Sino Longitudinal Study on Cognitive Decline
STT-A: Shape Trails Test Part A
STT-B: Shape Trails Test Part B
SUVR: Standard uptake value rate

## References

[1] Jia J, Wei C, Chen S, Li F, Tang Y, Qin W (2018). The cost of Alzheimer's disease in China and re-estimation of costs worldwide. Alzheimers Dement.

[2] Jack CR, Bennett DA, Blennow K, Carrillo MC, Dunn B, Haeberlein SB (2018). NIA-AA research framework: toward a biological definition of Alzheimer's disease. Alzheimers Dement.

[3] Wang X, Huang W, Su L, Xing Y, Jessen F, Sun Y (2020). Neuroimaging advances regarding subjective cognitive decline in preclinical Alzheimer's disease. Mol Neurodegener.

[4] Scheltens P, Blennow K, Breteler MM, de Strooper B, Frisoni GB, Salloway S (2016). Alzheimer's disease. Lancet..

[5] Cryan JF, O'Riordan KJ, Sandhu K, Peterson V, Dinan TG (2020). The gut microbiome in neurological disorders. Lancet Neurol.

[6] Cryan JF, O'Riordan KJ, Cowan CSM, Sandhu KV, Bastiaanssen TFS, Boehme M (2019). The microbiota-gut-brain axis. Physiol Rev.

[7] Sherwin E, Dinan TG, Cryan JF (2018). Recent developments in understanding the role of the gut microbiota in brain health and disease. Ann N Y Acad Sci.

[8] Cattaneo A, Cattane N, Galluzzi S, Provasi S, Lopizzo N, Festari C (2017). Association of brain amyloidosis with pro-inflammatory gut bacterial taxa and peripheral inflammation markers in cognitively impaired elderly. Neurobiol Aging.

[9] Abraham D, Feher J, Scuderi GL, Szabo D, Dobolyi A, Cservenak M (2019). Exercise and probiotics attenuate the development of Alzheimer's disease in transgenic mice: role of microbiome. Exp Gerontol.

[10] Bonfili L, Cecarini V, Berardi S, Scarpona S, Suchodolski JS, Nasuti C (2017). Microbiota modulation counteracts Alzheimer's disease progression influencing neuronal proteolysis and gut hormones plasma levels. Sci Rep.

[11] Liu P, Wu L, Peng G, Han Y, Tang R, Ge J (2019). Altered microbiomes distinguish Alzheimer's disease from amnestic mild cognitive impairment and health in a Chinese cohort. Brain Behav Immun.

[12] Vogt NM, Kerby RL, Dill-McFarland KA, Harding SJ, Merluzzi AP, Johnson SC (2017). Gut microbiome alterations in Alzheimer's disease. Sci Rep.

[13] Li B, He Y, Ma J, Huang P, Du J, Cao L (2019). Mild cognitive impairment has similar alterations as Alzheimer's disease in gut microbiota. Alzheimers Dement.

[14] Sheng C, Lin L, Lin H, Wang X, Han Y, Liu SL (2021). Altered gut microbiota in adults with subjective cognitive decline: the SILCODE study. J Alzheimer's Dis..

[15] Chouraki V, Beiser A, Younkin L, Preis SR, Weinstein G, Hansson O (2015). Plasma amyloid-β and risk of Alzheimer's disease in the Framingham Heart Study. Alzheimers Dement.

[16] Hanon O, Vidal JS, Lehmann S, Bombois S, Allinquant B, Tréluyer JM (2018). Plasma amyloid levels within the Alzheimer's process and correlations with central biomarkers. Alzheimers Dement.

[17] Song F, Poljak A, Valenzuela M, Mayeux R, Smythe GA, Sachdev PS (2011). Meta-analysis of plasma amyloid-β levels in Alzheimer's disease. J Alzheimer's Dis.

[18] Lim YY, Maruff P, Kaneko N, Doecke J, Fowler C, Villemagne VL (2020). Plasma amyloid-β biomarker associated with cognitive decline in preclinical Alzheimer's disease. J Alzheimer's Dis.

[19] Nakamura A, Kaneko N, Villemagne VL, Kato T, Doecke J, Doré V (2018). High performance plasma amyloid-β biomarkers for Alzheimer's disease. Nature..

[20] Pérez-Grijalba V, Arbizu J, Romero J, Prieto E, Pesini P, Sarasa L (2019). Plasma Aβ42/40 ratio alone or combined with FDG-PET can accurately predict amyloid-PET positivity: a cross-sectional analysis from the AB255 Study. Alzheimers Res Ther.

[21] Li X, Wang X, Su L, Hu X, Han Y (2019). Sino Longitudinal Study on Cognitive Decline (SILCODE): protocol for a Chinese longitudinal observational study to develop risk prediction models of conversion to mild cognitive impairment in individuals with subjective cognitive decline. BMJ Open.

[22] Bondi MW, Edmonds EC, Jak AJ, Clark LR, Delano-Wood L, McDonald CR (2014). Neuropsychological criteria for mild cognitive impairment improves diagnostic precision, biomarker associations, and progression rates. J Alzheimer's Dis.

[23] McKhann GM, Knopman DS, Chertkow H, Hyman BT, Jack CR, Kawas CH (2011). The diagnosis of dementia due to Alzheimer's disease: recommendations from the National Institute on Aging-Alzheimer's Association workgroups on diagnostic guidelines for Alzheimer's disease. Alzheimers Dement.

[24] Zhao Q, Lv Y, Zhou Y, Hong Z, Guo Q (2012). Short-term delayed recall of auditory verbal learning test is equivalent to long-term delayed recall for identifying amnestic mild cognitive impairment. PLoS One.

[25] Zhao Q, Guo Q, Li F, Zhou Y, Wang B, Hong Z (2013). The Shape Trail Test: application of a new variant of the Trail making test. PLoS One.

[26] Guo QJL, Hong Z, Lv C (2007). A specific phenomenon of animal fluency test in chinese elderly. Chin Mental Health J.

[27] Guo QHZ, Shi W, Sun Y, Lv C (2006). Boston naming test in Chinese elderly, patient with mild cognitive impairment and Alzheimer's dementia. Chin Mental Health J.

[28] Chen KL, Xu Y, Chu AQ, Ding D, Liang XN, Nasreddine ZS (2016). Validation of the Chinese version of montreal cognitive assessment basic for screening mild cognitive impairment. J Am Geriatr Soc.

[29] Qian Y, Yang X, Xu S, Wu C, Song Y, Qin N (2018). Alteration of the fecal microbiota in Chinese patients with Parkinson’s disease. Brain Behav Immun.

[30] Rognes T, Flouri T, Nichols B, Quince C, Mahé F (2016). VSEARCH: a versatile open source tool for metagenomics. PeerJ..

[31] Edgar RC (2010). Search and clustering orders of magnitude faster than BLAST. Bioinformatics..

[32] Chen K, Roontiva A, Thiyyagura P, Lee W, Liu X, Ayutyanont N (2015). Improved power for characterizing longitudinal amyloid-β PET changes and evaluating amyloid-modifying treatments with a cerebral white matter reference region. J Nucl Med.

[33] Li TR, Wu Y, Jiang JJ, Lin H, Han CL, Jiang JH (2020). Radiomics analysis of magnetic resonance imaging facilitates the identification of preclinical Alzheimer's Disease: An Exploratory study. Front Cell Dev Biol.

[34] DeLong ER, DeLong DM, Clarke-Pearson DL (1988). Comparing the areas under two or more correlated receiver operating characteristic curves: a nonparametric approach. Biometrics..

[35] Lövheim H, Elgh F, Johansson A, Zetterberg H, Blennow K, Hallmans G (2017). Plasma concentrations of free amyloid β cannot predict the development of Alzheimer's disease. Alzheimers Dement.

[36] Rembach A, Faux NG, Watt AD, Pertile KK, Rumble RL, Trounson BO (2014). Changes in plasma amyloid beta in a longitudinal study of aging and Alzheimer's disease. Alzheimers Dement.

[37] Olsson B, Lautner R, Andreasson U, Öhrfelt A, Portelius E, Bjerke M (2016). CSF and blood biomarkers for the diagnosis of Alzheimer's disease: a systematic review and meta-analysis. Lancet Neurol.

[38] Verberk IMW, Thijssen E, Koelewijn J, Mauroo K, Vanbrabant J, de Wilde A (2020). Combination of plasma amyloid beta((1-42/1-40)) and glial fibrillary acidic protein strongly associates with cerebral amyloid pathology. Alzheimers Res Ther.

[39] Bhat MI, Kapila R (2017). Dietary metabolites derived from gut microbiota: critical modulators of epigenetic changes in mammals. Nutr Rev.

[40] Kumar H, Lund R, Laiho A, Lundelin K, Ley RE, Isolauri E, et al. Gut microbiota as an epigenetic regulator: pilot study based on whole-genome methylation analysis. mBio. 2014;5:e02113–14. 10.1128/mBio.02113-14 PMC4271550 25516615

[41] Megur A, Baltriukienė D, Bukelskienė V, Burokas A. The microbiota-gut-brain axis and Alzheimer's disease: neuroinflammation is to blame? Nutrients. 2020;13:37. 10.3390/nu13010037 PMC7824474 33374235

[42] Hooper LV, Littman DR, Macpherson AJ (2012). Interactions between the microbiota and the immune system. Science..

[43] Goyal D, Ali SA, Singh RK (2021). Emerging role of gut microbiota in modulation of neuroinflammation and neurodegeneration with emphasis on Alzheimer's disease. Prog Neuropsychopharmacol Biol Psychiatry.

[44] Welcome MO (2019). Gut microbiota disorder, gut epithelial and blood-brain barrier dysfunctions in etiopathogenesis of dementia: molecular mechanisms and signaling pathways. Neuromolecular Med.

[45] Zhang L, Wang Y, Xiayu X, Shi C, Chen W, Song N (2017). Altered gut microbiota in a mouse model of Alzheimer's disease. J Alzheimer's Dis.

[46] Canfora EE, Jocken JW, Blaak EE (2015). Short-chain fatty acids in control of body weight and insulin sensitivity. Nat Rev Endocrinol.

[47] Morrison DJ, Preston T (2016). Formation of short chain fatty acids by the gut microbiota and their impact on human metabolism. Gut Microbes.

[48] Sanna S, van Zuydam NR, Mahajan A, Kurilshikov A, Vich Vila A, Võsa U (2019). Causal relationships among the gut microbiome, short-chain fatty acids and metabolic diseases. Nat Genet.

[49] Silva YP, Bernardi A, Frozza RL (2020). The role of short-chain fatty acids from gut microbiota in gut-brain communication. Front Endocrinol (Lausanne).

[50] Braniste V, Al-Asmakh M, Kowal C, Anuar F, Abbaspour A, Tóth M (2014). The gut microbiota influences blood-brain barrier permeability in mice. Sci Transl Med.

[51] Allin KH, Tremaroli V, Caesar R, Jensen BAH, Damgaard MTF, Bahl MI (2018). Aberrant intestinal microbiota in individuals with prediabetes. Diabetologia..

[52] Zhuang ZQ, Shen LL, Li WW, Fu X, Zeng F, Gui L (2018). Gut Microbiota is altered in patients with Alzheimer's disease. J Alzheimer's Dis.

[53] Rajilić-Stojanović M, de Vos WM (2014). The first 1000 cultured species of the human gastrointestinal microbiota. FEMS Microbiol Rev.

[54] Zhan X, Stamova B, Sharp FR (2018). Lipopolysaccharide associates with amyloid plaques, neurons and oligodendrocytes in Alzheimer's disease brain: a review. Front Aging Neurosci.

[55] Lukiw WJ (2016). Bacteroides fragilis lipopolysaccharide and inflammatory signaling in Alzheimer's disease. Front Microbiol.

[56] Waite DW, Chuvochina M, Pelikan C, Parks DH, Yilmaz P, Wagner M (2020). Proposal to reclassify the proteobacterial classes Deltaproteobacteria and Oligoflexia, and the phylum Thermodesulfobacteria into four phyla reflecting major functional capabilities. Int J Syst Evol Microbiol.

[57] Attene-Ramos MS, Wagner ED, Plewa MJ, Gaskins HR (2006). Evidence that hydrogen sulfide is a genotoxic agent. Mol Cancer Res.

[58] Sheng C, Sun Y, Wang M, Wang X, Liu Y, Pang D (2020). Combining visual rating scales for medial temporal lobe atrophy and posterior atrophy to identify amnestic mild cognitive impairment from cognitively normal older adults: evidence based on two cohorts. J Alzheimer's Dis.

[59] Harper L, Fumagalli GG, Barkhof F, Scheltens P, O'Brien JT, Bouwman F (2016). MRI visual rating scales in the diagnosis of dementia: evaluation in 184 post-mortem confirmed cases. Brain..

[60] Liu YX, Qin Y, Chen T, Lu M, Qian X, Guo X (2021). A practical guide to amplicon and metagenomic analysis of microbiome data. Protein Cell..

