# Supplementary material for: Combination of gut microbiota and plasma amyloid-β as a potential index for identifying preclinical Alzheimer’s disease: a cross-sectional analysis from the SILCODE study
Source: Alzheimers Res Ther. 2022 Feb 14;14:35. doi: 10.1186/s13195-022-00977-x (PMC8843023; doi:10.1186/s13195-022-00977-x)
Supplement: Supplementary file 1 — Additional file 1 : Supplementary Table S1. The items of the semi-structured interview for lifestyles. Supplementary Table S2. Demographic information and neuropsychological assessments for CI patients. Supplementary Table S3. Gut microbial differences between CN- and CN+ with correction for multiple comparisons. Supplementary Table S4. The interaction effect between APOE and diagnosis in the altered gut microbiota for CN- and CN+ participants. Supplementary Figure S1. The alpha diversity of gut microbiota among the CN-, CN+, and CI groups. Each bar graph represented the mean and standard deviation. The Chao1 and ACE indexes showed significantly decline in CI compared with CN-. There were no significant differences in Simpson and Shannon indexes among the three groups. *, p < 0.05. CN-, amyloid-β negative cognitively normal participants; CN+, amyloid-β positive cognitively normal participants; CI, cognitive impairment participants. Supplementary Figure S2. The PCoA and NMDS based on the distribution of ASVs. (A) PCoA showed that the difference of gut taxonomic composition between CN- and CI was marginal in statistical significance (F = 1.383, p < 0.052); (B) NMDS showed that the gut taxonomic composition was significantly different between CN- and CI (R = 0.097, p < 0.009); (C) PCoA showed that the gut taxonomic composition between CN+ and CI was not significantly different (F = 0.850, p < 0.712); (D) NMDS showed that the structure of gut microbiota in the CN+ group was not significantly different from that in the CI group (F = -0.020, p < 0.698). CN-, amyloid-β negative cognitively normal participants; CN+, amyloid-β positive cognitively normal participants; CI, cognitive impairment participants; ASV, amplicon sequence variants; PCoA, principal coordinates analysis; PERMANOVA, permutational multivariate analysis of variance; NMDS, non-metric multidimensional scaling; ANOSIM, analysis of similarities. Supplementary Figure S3. The relative abundance of altered gut mi [file 13195_2022_977_MOESM1_ESM.zip › Supplementary Appendix.docx]

**Combination of gut microbiota and plasma amyloid-β as a potential index for identifying preclinical Alzheimer’s disease: a cross-sectional analysis from the SILCODE study**

**Supplementary Methods**

**Sample size calculation**

According to a previous study of our group [1], the mean relative abundance (standard deviation) of the predominant phylum *Firmicutes* in CI patients and normal controls were 30.88 (12.91) and 49.53 (15.74), respectively. We estimated that the effect size was about 1.0. We also assumed that the power (1-β) was 80%, and ɑ was 0.05 in our study. Thus, the required total sample size was 36 (per group is 18). In this study, we recruited 34 individuals with CN- and 32 group-matched CN+ (n = 66).

**Reference**

[1] Sheng C, Lin L, Lin H, Wang X, Han Y, Liu SL. Altered Gut Microbiota in Adults with Subjective Cognitive Decline: The SILCODE Study. J Alzheimers Dis. 2021;82(2):513-526. doi: 10.3233/JAD-210259.

**Tables**

**Supplementary Table S1.** The items of the semi-structured interview for lifestyles

| **Lifestyles items** |  |  |
| --- | --- | --- |
| **Diet** | Carnivorous diet:  (1) red meat; (2) chicken; (3) fish; (4) others | 1. Eating meat frequency:  (1) every day; (2) > 3 times/week; (3) < 3 times/week; (4) occasionally; (5) no  2. Eating vegetables frequency:  (1) every day; (2) > 3 times/week; (3) < 3 times/week; (4) occasionally; (5) no  3. Eating fruits frequency:  (1) every day; (2) > 3 times/week; (3) < 3 times/week; (4) occasionally; (5) no  4. Eating sweetmeats frequency:  (1) every day; (2) > 3 times/week; (3) < 3 times/week; (4) occasionally; (5) no  5. Eating grain frequency:  (1) every day; (2) > 3 times/week; (3) < 3 times/week; (4) occasionally; (5) no  6. Carbonated drink frequency:  (1) every day; (2) > 3 times/week; (3) < 3 times/week; (4) occasionally; (5) no |
|  | Vegetarian diet |  |
|  | General diet |  |
| **Alcohol** | Baijiu | 1. Alcohol consumption: units/day  2. Drinking frequency:  (1) every day; (2) > 3 times/week; (3) < 3 times/week; (4) occasionally; (5) no  3. Quit drinking:  (1) yes; (2) no |
|  | Red wine |  |
|  | Beer |  |
|  | Others |  |
|  | No drinking |  |
| **Smoking** | Whether smoking:  (1) yes; (2) no | Quit smoking:  (1) yes; (2) no |
| **Exercise** | Aerobics | Exercise frequency: (1) every day; (2) > 3 times/week; (3) < 3 times/week; (4) occasionally; (5) no |
|  | Anaerobic exercise |  |

**Supplementary Table S2.** Demographic information and neuropsychological assessments for CI patients

|  | **Total CI (n = 22)** | **Stratified for diagnosis** | |
| --- | --- | --- | --- |
|  |  | **MCI (n = 11)** | **AD (n = 11)** |
| **Demographic information** |  |  |  |
| Age (years) | 74.41 ± 7.49 | 72.27 ± 6.36 | 76.55 ± 8.20 |
| Sex (M/F) | 5/17 | 1/10 | 4/7 |
| Education (years) | 10.09 ± 5.05 | 9.00 ± 5.04 | 11.18 ± 5.06 |
| BMI | 23.24 ± 3.17 | 23.06 ± 3.42 | 23.42 ± 3.06 |
| APOE ε4 (%) ^a^ | 7 (41.18%) | 4 (44.44%) | 3 (37.5%) |
| Diabetes (%) | 5 (22.73%) | 3 (27.27%) | 2 (18.18%) |
| Hypertension (%) | 11 (50%) | 5 (45.45%) | 6 (54.55%) |
| **Neuropsychological tests** |  |  |  |
| HAMD | 4.77 ± 6.65 | 6.45 ± 8.96 | 3.09 ± 2.55 |
| HAMA | 5.14 ± 6.21 | 6.27 ± 8.28 | 4.00 ± 3.10 |
| MoCA-B | 15.14 ± 5.63 | 19.00 ± 4.02 | 11.27 ± 4.17 |
| AVLT-D (long) | 0.91 ± 1.48 | 1.82 ± 1.66 | 0 |
| AVLT-R | 15.05 ± 4.93 | 18.00 ± 2.45 | 12.09 ± 5.09 |
| STT-A ^b^ | 116.62 ± 51.90 | 100.55 ± 43.15 | 134.30 ± 57.04 |
| STT-B | 256.95 ± 84.99 | 229.09 ± 79.93 | 287.60 ± 83.43 |
| AFT | 10.82 ± 4.79 | 13.64 ± 4.80 | 8.00 ± 2.76 |
| BNT | 18.50 ± 4.36 | 19.73 ± 4.03 | 17.27 ± 4.52 |
| FAQ | 9.36 ± 7.25 | 4.36 ± 3.93 | 14.36 ± 6.33 |

Notes: a, only nine MCI patients and eight AD patients had APOE data; b, one AD patient without STT-A score. Abbreviations: CI, cognitive impairment participants; MCI, mild cognitive impairment; AD, Alzheimer’s dementia; M, male; F, female; BMI, body mass index; APOE, apolipoprotein E; HAMD, Hamilton Depression Rating Scale; HAMA, Hamilton Anxiety Rating Scale; MoCA-B, Montreal Cognitive Assessment-Basic version; AVLT-D (long), Auditory Verbal Learning Test-long delayed recall; AVLT-R, Auditory Verbal Learning Test-recognition; STT-A, Shape Trails Test Part A; STT-B, Shape Trails Test Part B; AFT, Animal Fluency Test; BNT, Boston Naming Test; FAQ, Functional Activities Questionnaire.

**Supplementary Table S3.** Gut microbial differences between CN- and CN+ with correction for multiple comparisons

|  | **CN- (n = 34)** | **CN+ (n = 32)** | ***P* value** | ***Corrected P* value** |
| --- | --- | --- | --- | --- |
| p_Bacteroidetes | 40.01 ± 16.14 | 50.76 ± 18.25 | 0.014 | **0.042** |
| p_Firmicutes | 51.85 ± 16.08 | 39.71 ± 17.26 | 0.004 | **0.012** |
| c_Bacteroidia | 40.00 ± 16.15 | 50.76 ± 18.25 | 0.014 | **0.042** |
| c_Clostridia | 42.93 ± 15.01 | 32.26 ± 16.71 | 0.009 | **0.045** |
| c_Deltaproteobacteria | 0.39 ± 0.51 | 0.15 ± 0.25 | 0.002 | **0.010** |
| o_Bacteroidales | 40.00 ± 16.15 | 50.76 ± 18.25 | 0.014 | 0.070 |
| o_Clostridiales | 42.93 ± 15.01 | 32.26 ± 16.71 | 0.009 | **0.045** |
| o_Desulfovibrionales | 0.39 ± 0.51 | 0.15 ± 0.25 | 0.002 | **0.010** |
| f_Lachnospiraceae | 21.92 ± 9.50 | 16.33 ± 7.19 | 0.006 | 0.066 |
| f_Ruminococcaceae | 18.86 ± 11.79 | 12.93 ± 11.82 | 0.029 | 0.319 |
| f_Desulfovibrionaceae | 0.39 ± 0.51 | 0.15 ± 0.25 | 0.002 | **0.022** |
| g_Faecalibacterium | 11.41 ± 8.65 | 7.08 ± 7.99 | 0.020 | 0.340 |

**Supplementary Table S4.** The interaction effect between APOE and diagnosis in the altered gut microbiota for CN- and CN+ participants

|  | CN- | | CN+ | | F (1, 62) | *P* value |
| --- | --- | --- | --- | --- | --- | --- |
|  | APOE ε4 carrier  (n = 11) | APOE ε4 non-carrier  (n = 23) | APOE ε4 carrier  (n = 12) | APOE ε4  non-carrier  (n = 20) |  |  |
| p_Bacteroidetes | 39.53 ± 20.79 | 40.23 ± 13.94 | 53.44 ± 19.84 | 49.16 ± 17.56 | 0.306 | 0.582 |
| p_Firmicutes | 53.13 ± 18.33 | 51.24 ± 15.30 | 38.55 ± 18.82 | 40.41 ± 16.73 | 0.184 | 0.670 |
| c_Bacteroidia | 39.53 ± 20.79 | 40.22 ± 13.95 | 53.44 ± 19.84 | 49.15 ± 17.56 | 0.305 | 0.583 |
| c_Clostridia | 45.83 ± 16.98 | 41.54 ± 14.16 | 28.02 ± 15.81 | 34.81 ± 17.10 | 1.820 | 0.182 |
| c_Deltaproteobacteria | 0.18 ± 0.27 | 0.48 ± 0.57 | 0.07 ± 0.11 | 0.20 ± 0.31 | 0.705 | 0.404 |
| o_Bacteroidales | 39.53 ± 20.79 | 40.22 ± 13.95 | 53.44 ± 19.84 | 49.15 ± 17.56 | 0.305 | 0.583 |
| o_Clostridiales | 45.83 ± 16.98 | 41.54 ± 14.16 | 28.02 ± 15.81 | 34.81 ± 17.10 | 1.820 | 0.182 |
| o_Desulfovibrionales | 0.18 ± 0.27 | 0.48 ± 0.57 | 0.07 ± 0.11 | 0.20 ± 0.31 | 0.705 | 0.404 |
| f_Lachnospiraceae | 24.01 ± 12.38 | 20.93 ± 7.90 | 14.79 ± 4.41 | 17.26 ± 8.41 | 1.600 | 0.211 |
| f_Ruminococcaceae | 19.04 ± 11.31 | 18.77 ± 12.25 | 11.25 ± 12.93 | 13.93 ± 11.32 | 0.228 | 0.635 |
| f_Desulfovibrionaceae | 0.18 ± 0.27 | 0.48 ± 0.57 | 0.07 ± 0.11 | 0.20 ± 0.31 | 0.705 | 0.404 |
| g_Bilophila | 0.10 ± 0.11 | 0.37 ± 0.58 | 0.06 ± 0.10 | 0.17 ± 0.28 | 0.748 | 0.390 |
| g_Faecalibacterium | 11.78 ± 8.71 | 11.23 ± 8.81 | 6.60 ± 7.49 | 7.36 ± 8.46 | 0.089 | 0.766 |

Abbreviations: CN-, amyloid-β negative cognitively normal participants; CN+, amyloid-β positive cognitively normal participants; APOE, apolipoprotein E; p, phylum; c, class; o, order; f, family; g, genus.

**Figure Legend**

**Supplementary Fig. S1** The alpha diversity of gut microbiota among the CN-, CN+, and CI groups. Each bar graph represented the mean and standard deviation. The Chao1 and ACE indexes showed significantly decline in CI compared with CN-. There were no significant differences in Simpson and Shannon indexes among the three groups. *, p < 0.05. CN-, amyloid-β negative cognitively normal participants; CN+, amyloid-β positive cognitively normal participants; CI, cognitive impairment participants.

**Supplementary Fig. S2** The PCoA and NMDS based on the distribution of ASVs. (A) PCoA showed that the difference of gut taxonomic composition between CN- and CI was marginal in statistical significance (F = 1.383, p < 0.052); (B) NMDS showed that the gut taxonomic composition was significantly different between CN- and CI (R = 0.097, p < 0.009); (C) PCoA showed that the gut taxonomic composition between CN+ and CI was not significantly different (F = 0.850, p < 0.712); (D) NMDS showed that the structure of gut microbiota in the CN+ group was not significantly different from that in the CI group (F = -0.020, p < 0.698). CN-, amyloid-β negative cognitively normal participants; CN+, amyloid-β positive cognitively normal participants; CI, cognitive impairment participants; ASV, amplicon sequence variants; PCoA, principal coordinates analysis; PERMANOVA, permutational multivariate analysis of variance; NMDS, non-metric multidimensional scaling; ANOSIM, analysis of similarities.

**Supplementary Fig. S3** The relative abundance of altered gut microbiota at different taxonomic levels among the CN-, CN+ and CI groups. Bar graphs indicated the relative abundance of phylum-level (A), class-level (B), order-level (C), family-level (D), and genus-level (E) taxa. The phylum *Firmicutes* and its corresponding class *Clostridia*, order *Clostridiales*, family *Desulfovibrionaceae*, family *Ruminococcaceae*, genus *Lachnospiracea_incertae_sedis* and genus *Faecalibacterium* taxa showed a progressive decline from CN- to CN+ and CI. CN-, amyloid-β negative cognitively normal participants; CN+, amyloid-β positive cognitively normal participants; CI, cognitive impairment participants.
