# Supplementary figures and images for: Combination of gut microbiota and plasma amyloid-β as a potential index for identifying preclinical Alzheimer’s disease: a cross-sectional analysis from the SILCODE study
Source: Alzheimers Res Ther. 2022 Feb 14;14:35. doi: 10.1186/s13195-022-00977-x (PMC8843023; doi:10.1186/s13195-022-00977-x)

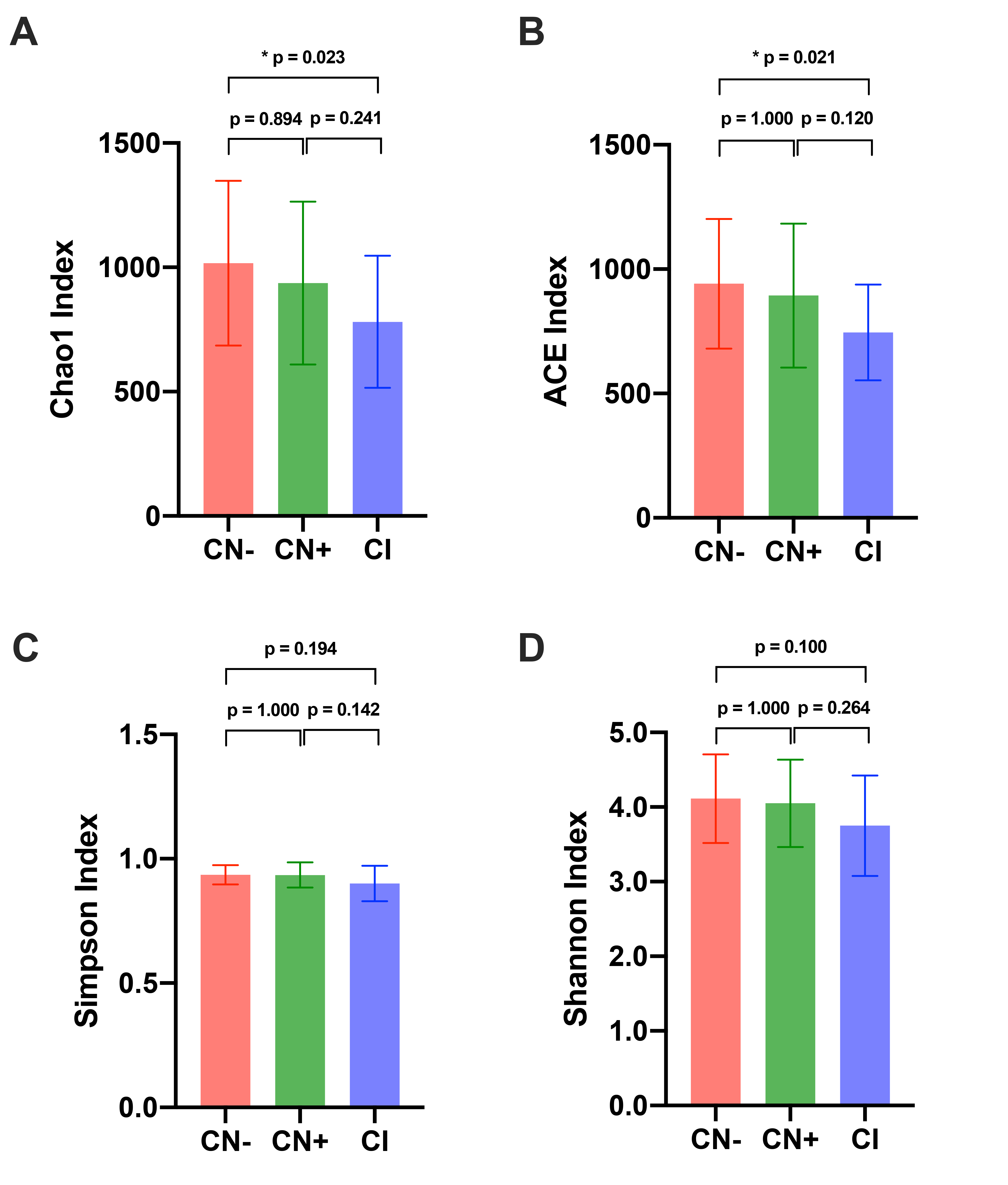

Supplement: Supplementary file 1 — Additional file 1 : Supplementary Table S1. The items of the semi-structured interview for lifestyles. Supplementary Table S2. Demographic information and neuropsychological assessments for CI patients. Supplementary Table S3. Gut microbial differences between CN- and CN+ with correction for multiple comparisons. Supplementary Table S4. The interaction effect between APOE and diagnosis in the altered gut microbiota for CN- and CN+ participants. Supplementary Figure S1. The alpha diversity of gut microbiota among the CN-, CN+, and CI groups. Each bar graph represented the mean and standard deviation. The Chao1 and ACE indexes showed significantly decline in CI compared with CN-. There were no significant differences in Simpson and Shannon indexes among the three groups. *, p < 0.05. CN-, amyloid-β negative cognitively normal participants; CN+, amyloid-β positive cognitively normal participants; CI, cognitive impairment participants. Supplementary Figure S2. The PCoA and NMDS based on the distribution of ASVs. (A) PCoA showed that the difference of gut taxonomic composition between CN- and CI was marginal in statistical significance (F = 1.383, p < 0.052); (B) NMDS showed that the gut taxonomic composition was significantly different between CN- and CI (R = 0.097, p < 0.009); (C) PCoA showed that the gut taxonomic composition between CN+ and CI was not significantly different (F = 0.850, p < 0.712); (D) NMDS showed that the structure of gut microbiota in the CN+ group was not significantly different from that in the CI group (F = -0.020, p < 0.698). CN-, amyloid-β negative cognitively normal participants; CN+, amyloid-β positive cognitively normal participants; CI, cognitive impairment participants; ASV, amplicon sequence variants; PCoA, principal coordinates analysis; PERMANOVA, permutational multivariate analysis of variance; NMDS, non-metric multidimensional scaling; ANOSIM, analysis of similarities. Supplementary Figure S3. The relative abundance of altered gut mi [file 13195_2022_977_MOESM1_ESM.zip › Figure S1.tif]

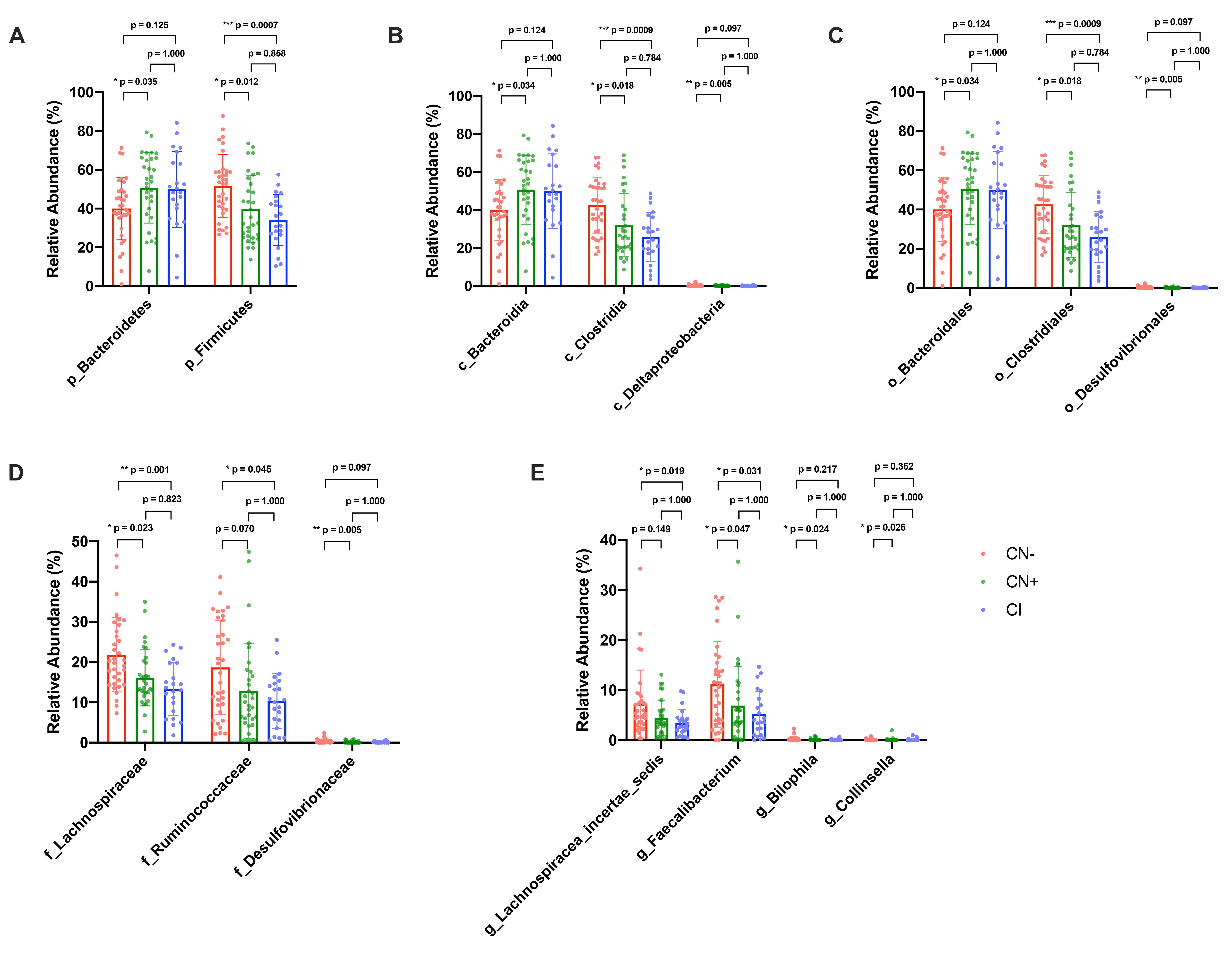

Supplement: Supplementary file 1 — Additional file 1 : Supplementary Table S1. The items of the semi-structured interview for lifestyles. Supplementary Table S2. Demographic information and neuropsychological assessments for CI patients. Supplementary Table S3. Gut microbial differences between CN- and CN+ with correction for multiple comparisons. Supplementary Table S4. The interaction effect between APOE and diagnosis in the altered gut microbiota for CN- and CN+ participants. Supplementary Figure S1. The alpha diversity of gut microbiota among the CN-, CN+, and CI groups. Each bar graph represented the mean and standard deviation. The Chao1 and ACE indexes showed significantly decline in CI compared with CN-. There were no significant differences in Simpson and Shannon indexes among the three groups. *, p < 0.05. CN-, amyloid-β negative cognitively normal participants; CN+, amyloid-β positive cognitively normal participants; CI, cognitive impairment participants. Supplementary Figure S2. The PCoA and NMDS based on the distribution of ASVs. (A) PCoA showed that the difference of gut taxonomic composition between CN- and CI was marginal in statistical significance (F = 1.383, p < 0.052); (B) NMDS showed that the gut taxonomic composition was significantly different between CN- and CI (R = 0.097, p < 0.009); (C) PCoA showed that the gut taxonomic composition between CN+ and CI was not significantly different (F = 0.850, p < 0.712); (D) NMDS showed that the structure of gut microbiota in the CN+ group was not significantly different from that in the CI group (F = -0.020, p < 0.698). CN-, amyloid-β negative cognitively normal participants; CN+, amyloid-β positive cognitively normal participants; CI, cognitive impairment participants; ASV, amplicon sequence variants; PCoA, principal coordinates analysis; PERMANOVA, permutational multivariate analysis of variance; NMDS, non-metric multidimensional scaling; ANOSIM, analysis of similarities. Supplementary Figure S3. The relative abundance of altered gut mi [file 13195_2022_977_MOESM1_ESM.zip › Figure S3.tif]
